# Multi-Omics Integration Identifies the Cholesterol Metabolic Enzyme *DHCR24* as a Key Driver in Breast Cancer

**DOI:** 10.3390/biology15010040

**Published:** 2025-12-25

**Authors:** Mingfei Xu, Jinghua Hu, Lulan Pu, Jiayou Liu, Yanhong Yang, Qianqian Li, Jingwen Chen, Shishan Deng, Chaoyue Liu

**Affiliations:** Institute of Basic Medicine, North Sichuan Medical College, Nanchong 637000, China

**Keywords:** breast cancer, cholesterol metabolism, *DHCR24*, multi-omics, mendelian randomization, therapeutic target, bioinformatics

## Abstract

Dysregulated cholesterol metabolism is a hallmark of breast cancer (BC), but its key molecular mediators remain unclear. Using an integrated multi-omics approach, including Mendelian randomization, transcriptomic/proteomic database screening, functional assays, and clinical correlation, we identified the cholesterol biosynthesis enzyme *DHCR24* as a central metabolic-immune mediator. We found that high *DHCR24* mRNA expression is associated with poorer patient prognosis and is elevated in luminal and HER2+ subtypes. Surprisingly, *DHCR24* knockdown enhanced malignant phenotypes in MCF7 cells, contrasting its pro-tumor role in patients. Integrated analysis resolved this paradox, revealing that *DHCR24* promotes BC progression non-cell-autonomously by remodeling an immunosuppressive tumor microenvironment, rather than by intrinsically driving cancer cell proliferation. Mechanistically, *DHCR24* depletion upregulated *TP53* and downregulated *SQLE*. This study establishes *DHCR24* as a pivotal metabolic-immune node and a promising therapeutic target for disrupting the cholesterol–immune axis in luminal and HER2+ BC.

## Simple Summary

Scientists have long suspected a link between cholesterol and breast cancer, but proving a direct cause was difficult. Our research used genetic analysis to confirm that high cholesterol levels can indeed increase the risk of developing breast cancer. We then identified a key gene, *DHCR24*, which is involved in cholesterol production and is often found at high levels in breast cancer patients. We discovered that this gene does more than just manage cholesterol; it primarily helps the cancer by creating a protective shield that blocks the body’s immune cells from attacking the tumor. This finding helps explain why drugs that lower cholesterol might be beneficial and points to *DHCR24* as a promising new target for treatment. Our work suggests that future therapies focusing on this gene could potentially weaken the tumor’s defenses and make it more vulnerable to the immune system, offering new hope for personalized breast cancer care.

## 1. Introduction

Breast cancer (BC) continues to pose a significant global health challenge, with over 2.3 million new cases diagnosed in 2022 alone [1]. Despite advancements in standard therapies—including chemotherapy, targeted agents, and more recently, immunotherapy—treatment outcomes remain suboptimal for many patients. Limitations such as intrinsic or acquired therapeutic resistance, narrow applicability of immune checkpoint inhibitors, and high treatment costs underscore the urgent need for novel therapeutic strategies [2].

In recent years, tumor metabolic reprogramming has emerged as a hallmark of cancer [3]. To comprehensively decipher the complex metabolic drivers of cancer progression, integrated multi-omics approaches have proven invaluable, enabling the systematic identification of key molecular mediators [4,5]. Among these, dysregulated cholesterol metabolism gaining particular attention for its dual role in tumor biology [6,7]. Beyond its fundamental functions in maintaining membrane integrity and facilitating steroidogenesis, cholesterol actively contributes to malignant progression [8,9]. It influences membrane fluidity and signaling platform assembly, thereby modulating cancer cell motility, invasion, and metastasis [6]. Moreover, cholesterol and its metabolites can impair chemotherapeutic efficacy by reducing drug permeability and participate in broader cellular signaling networks [10]. However, the relationship between cholesterol and cancer risk is complex and context-dependent, exhibiting both pro-tumorigenic and protective effects across different cancer types and stages [11].

In BC, the role of cholesterol is particularly multifaceted and not fully understood [11]. A key challenge lies in the lack of systematic studies to identify the specific molecular mediators that link cholesterol metabolism to disease progression, particularly through the application of integrated multi-omics frameworks [4]. Among potential candidates, *DHCR24* (Δ24-sterol reductase, located on chromosome 1p32.3)—a critical enzyme in the cholesterol biosynthesis pathway—has drawn increasing interest in oncology [12]. Beyond its canonical metabolic function, *DHCR24* expression is subject to complex regulation, including potential epigenetic mechanisms such as promoter methylation, which has been implicated in its dysregulation in other malignancies [13]. *DHCR24* is frequently overexpressed in malignancies and is associated with poor prognosis [12,14]. It promotes tumor cell proliferation, migration, and invasion by stabilizing lipid rafts and activating oncogenic pathways such as *PI3K-AKT* and *Ras-Raf* [13,15,16]. *DHCR24* also exerts anti-apoptotic effects, partly through modulation of *p53* acetylation and subcellular localization [17,18]. In BC, *DHCR24* upregulation has been linked to the expansion of stem-like cell populations via Hedgehog signaling activation [16].

Despite these advances, several critical gaps persist, necessitating a novel, systematic perspective [12]. First, causal evidence linking systemic cholesterol levels to BC risk is limited [11]. Second, existing studies on *DHCR24* often focus on a single molecular layer (e.g., transcriptome or function), lacking an integrative view that connects genetic predisposition, multi-omics landscapes, experimental validation, and clinical outcome within a unified framework [12]. This fragmented approach limits a holistic understanding of *DHCR24*’s role as a potential metabolic-immune mediator.

To address these gaps, we employed a causality-anchored, multi-omics strategy designed to provide a systematic and novel view. Our approach uniquely integrates: (1) Mendelian randomization (MR) to establish a genetic causal link between total cholesterol and BC risk; (2) Comprehensive multi-omics screening across transcriptomic and proteomic dimensions to prioritize *DHCR24* within this causal context; (3) Functional validation of its role in malignant phenotypes; and (4) Mechanistic and clinical correlation analyses to elucidate its impact on signaling pathways and the tumor microenvironment. This study thereby moves beyond associative observations to delineate *DHCR24*’s role through a cohesive, multi-lens investigation, offering new insights into cholesterol-driven oncogenesis and identifying potential therapeutic targets for specific BC subtypes.

## 2. Materials and Methods

### 2.1. Overall Research Strategy

To systematically investigate the role of cholesterol metabolism in BC beyond associative links, we employed a unique, causality-anchored multi-phase framework. The strength of this strategy lies in its sequential integration of genetic epidemiology (to infer causality), multi-omics bioinformatics screening (to objectively prioritize a key mediator from public databases), in vitro functional validation (to establish biological mechanism), and clinical correlation (to assess translational relevance). This design provides a comprehensive and robust evidence chain that single-method approaches cannot achieve.

### 2.2. Study Design and Data Sources

#### 2.2.1. Two-Sample Mendelian Randomization

Data Source: in this study, we obtained the Genome-wide association study (GWAS) summary statistics for exposure (total cholesterol levels) and outcome (breast cancer [BC]) from the IEU OpenGWAS database (https://gwas.mrcieu.ac.uk/, accessed on 15 August 2024). This open-source database provides scalable, cloud-based infrastructure for publishing and accessing complete GWAS summary datasets and metadata [19]. The exposure was total cholesterol (dataset ID: met-d-Total_C; *n* = 115,078), and the outcome was BC (dataset ID: ieu-a-1131; *n* = 32,498 cases/controls).

Instrumental Variable Selection: To select instrumental variables (IVs), we identified single-nucleotide polymorphisms (SNPs) significantly associated with total cholesterol levels at the genome-wide significance threshold (*p* < 5 × 10^−8^). We applied linkage disequilibrium (LD) clumping (r^2^ < 0.01 within a 10,000 kb clumping distance) to ensure independence of the IVs. SNPs exhibiting potential pleiotropic effects on BC risk were excluded. The strength of the IVs was assessed by calculating the F-statistic. The mean F-statistic was 75.09, well above the conventional threshold of 10, indicating that weak instrument bias is unlikely. Ultimately, 24 independent SNPs met all these criteria and were included as IVs in the primary analysis.

Data Analysis: We performed a two-sample MR analysis using the R (version 4.2.1) package “TwoSampleMR”. The association between each selected IV and total cholesterol was quantified using GWAS summary statistics. The causal effect of total cholesterol levels on BC risk was estimated using the inverse-variance weighted (IVW) method.

Sensitivity Analysis: To assess robustness and potential biases, several sensitivity analyses were conducted. Heterogeneity among IV-specific causal estimates was evaluated using Cochran’s Q test. Horizontal pleiotropy was assessed through MR-Egger regression method. A ‘Leave-One-Out analysis’ was performed by iteratively removing individual SNPs to check if any single variant excessively influenced the overall causal estimate.

This MR analysis formed the foundational step to establish a causal link between the exposure (cholesterol) and the outcome (BC risk), prior to investigating downstream molecular mediators.

#### 2.2.2. Bioinformatics Analysis

**Candidate gene screening:** We used several bioinformatics databases to identify and characterize cholesterol-related genes potentially relevant to BC:

GeneCards (https://www.genecards.org/, accessed on 15 August 2023): To compile an initial gene list, we queried GeneCards and obtained its ranked list of the top 50 cholesterol-related genes. Genecards aggregates information from genomic, transcriptomic, proteomic, genetic, functional, and clinical sources [20].

GEPIA (http://gepia.cancer-pku.cn/, accessed on 15 August 2023): We then used GEPIA to compare expression levels of these 50 genes between BC and normal breast tissue. GEPIA leverages RNA-seq data from TCGA and GTEx [21].

Kaplan–Meier (KM) plotter (https://kmplot.com/analysis/index.php?p=service, accessed on 17 August 2023): To assess the prognostic significance of genes showing abnormal expression in BC, we analyzed their association with patient overall survival (OS) using the KM Plotter [22].

Human Protein Atlas (HPA) (https://www.proteinatlas.org/, accessed on 17 August 2023): Detailed protein expression and localization data for the selected cholesterol-related genes were retrieved from HPA, which includes extensive immunohistochemistry and omics data [23].

**Focus on *DHCR24*:** Based on the screening results, we conducted a deeper dive into the cholesterol biosynthesis gene *DHCR24*:

TCGA-BRCA RNA-seq: Data on *DHCR24* expression specifically in BC tissues (TCGA-BRCA dataset) was accessed directly via the TCGA portal (https://portal.gdc.cancer.gov/, accessed on 20 August 2023).

Human Protein Atlas (HPA): We retrieved the Immunohistochemical staining profile of *DHCR24* in BC tissue (https://www.proteinatlas.org/, accessed on 25 August 2023).

DepMap (https://depmap.org/portal/, accessed on 25 August 2023): We examined *DHCR24* expression levels across various BC cell lines utilizing the DepMap portal. We also explored data within DepMap to see how altering *DHCR24* (genetic perturbations) affects cell phenotypic effects in these cell lines [24].

TIMER 3.0 (https://compbio.cn/timer3/, accessed on 25 April 2025): We analyzed the association between *DHCR24* expression and immune infiltrates [25].

This systematic, multi-database bioinformatics screening was crucial to objectively prioritize *DHCR24* from numerous cholesterol-related genes based on its differential expression, prognostic value, and functional relevance, guiding our subsequent experimental focus.

### 2.3. In Vitro Experiments

#### 2.3.1. Immunohistochemical (IHC) Staining

IHC staining analysis was conducted on paraffin-embedded cancer and adjacent normal tissues from 59 treatment-naïve patients with non-specified invasive BC (2015–2018). All samples had complete clinicopathological records and no history of chemo/radiotherapy. Standard IHC for *DHCR24* involved dewaxing with xylene (Sigma-Aldrich, St. Louis, MO, USA), antigen retrieval using citrate buffer (pH 6.0) (Sigma-Aldrich, St. Louis, MO, USA), blocking endogenous peroxidase with 3% H_2_O_2_ (Sigma-Aldrich, St. Louis, MO, USA), incubating with the primary anti-DHCR24 antibody (Santa Cruz Biotechnology, Dallas, TX, USA) overnight at 4 °C, applying HRP-conjugated secondary antibody (Santa Cruz Biotechnology, Dallas, TX, USA) at room temperature for 1 h, DAB(TIAN GEN, Beijing, China) development, hematoxylin (Sigma-Aldrich, St. Louis, MO, USA) counterstaining, dehydration, and slide mounting. Staining results were evaluated according to the secondary staining intensity scoring criteria.

#### 2.3.2. Cell Culture

Human normal mammary epithelial cells (MCF10A) and BC cell lines (MCF7, MDAMB231, BT549, SKBR3, T47D) were purchased from Procell Biotechnology (Wuhan, China). All cell lines were maintained under recommended culture conditions (manufacturer-specified media, 37 °C with 5% CO_2_). Cells were passaged regularly when reaching approximately 80% confluence and tested routinely for mycoplasma contamination.

#### 2.3.3. Quantitative Real-Time PCR (qPCR)

Total RNA was extracted with TRIzol^®^ (Thermo Fisher Scientific, Carlsbad, CA, USA) and reverse-transcribed was carried out with the PrimeScript RT reagent Kit with gDNA Eraser (Takara Bio, Kusatsu, Shiga, Japan). SYBR Green-based qPCR (Takara Bio, Kusatsu, Shiga, Japan) was used for target gene detection with the following primer sequences:

for *β-actin* (100 bp), forward: 5′-GATCATTGCTCCTCCTGAGC-3′ and reverse: 5′-ACTCCTGCTTGCTGATCCAC-3′;

for *DHCR24* (297 bp), forward: 5′-CCCAGCGGCAGGAGAACCACTTCGT-3′ and reverse: 5′-ATCCAGCCAAAGAGGTAGCGGAAGA-3′;

for *P53* (193 bp), forward: 5′-AGCACTGTCCAACAACACCA-3′ and reverse: 5′-AGCACTGTCCAACAACACCA-3′;

for *SQLE* (142 bp), forward: 5′-GGTTACTCTGGTTACTGGGC-3′ and reverse: 5′-CTGCACTTCCCCACCGATAA-3′. The relative expression was calculated using the 2^−ΔCt^ method with *β-actin* as the reference gene. All qPCR experiments were designed, performed and reported in strict accordance with the MIQE guidelines [26,27]. This included validation of primer amplification efficiencies and confirmation of stable reference gene expression across experimental groups. All reactions were performed in technical duplicate for each of the three independent biological replicates.

#### 2.3.4. Western Blotting (WB)

Cell lysates were prepared, and proteins were separated by SDS-PAGE, then transferred onto PVDF membranes (Merck Millipore, Burlington, MA, USA). The membranes were blocked with 5% BSA (Sigma-Aldrich, St. Louis, MO, USA) in TBST, and incubated with primary antibodies (DHCR24 or EMT markers) (CST, Danvers, MA, USA) at 4 °C overnight. After that, the membranes were incubated with HRP-conjugated secondary and visualized using DAB reagent. Equal protein loading was confirmed by probing the same membrane for *β-actin*, which served as the loading control.

#### 2.3.5. Lentiviral Knockdown

MCF7 cells (3 × 10^5^ cells in 500 µL) were seeded in 24-well plates. Lentiviral particles targeting *DHCR24* and non-targeting control were purchased from Hanheng Biotechnology (Wuhan, China). Following an overnight incubation, the medium was replaced with fresh medium containing viral particles at a multiplicity of infection (MOI) of 30 (250 µL/well) and incubated for 72 h. When transduction efficiency (observed by fluorescence microscopy) reached approximately 90%, the cells were maintained in fresh medium with puromycin (3 µg/mL) (Sigma-Aldrich, St. Louis, MO, USA) for 14 d to select stably transduced cells. Knockdown efficiency was quantified at both the mRNA and protein levels. For mRNA quantification, qPCR was performed as described in Section 2.3.3. Briefly, total RNA was extracted, reverse transcribed, and amplified using gene-specific primers. For this specific experiment, the relative expression of *DHCR24* (normalized to *β-actin*) was calculated using the 2^−ΔCt^ method from three independent biological replicates, each measured in duplicate. A *p*-value < 0.05 was considered statistically significant.

For protein-level validation, Western blotting was carried out as detailed in Section 2.3.4. Membranes were stripped and re-probed for *β-actin* as the loading control. In this case, the intensity of *DHCR24* bands was quantified using ImageJ software v1.54g (Bethesda, MD, USA) and normalized to the corresponding *β-actin* signal. Data are derived from three independent biological replicates.

Statistical comparison between the *DHCR24*-KD group and the control group was performed using a two-tailed unpaired Student’s *t*-test. Data are presented as mean ± standard deviation (SD).

#### 2.3.6. Functional Assays

To experimentally validate the pro-tumorigenic role of *DHCR24* suggested by our bioinformatic analyses, MCF7 cells were divided into three groups. The first was the well plate control with just MCF7 cells. The second was the negative control group, in which MCF7 cells were infected with non-targeting lentivirus to ensure similar conditions except for the specific gene knockdown. The third was the experimental group, where *DHCR24*-specific lentivirus was used to reduce its expression. All groups were cultured under identical conditions to ensure the valid and reliable results.

Cell Proliferation (CCK-8 assay): The three groups of MCF7 cells were seeded into 96-well plates (100 µL/well containing 2000–5000 cells). After cell attachment, they were cultured for 24, 48, 72, and 96 h. At each time point, 10 µL/well of CCK-8 reagent (Dojindo, Germantown, MD, USA) was added. After incubating for 2 h at 37 °C, absorbance at 450 nm was measured with a microplate reader, and cell proliferation was plotted over time. This assay was performed to directly test the impact of *DHCR24* on a core hallmark of cancer—sustained proliferative signaling.

Cell apoptosis (Annexin V-APC/PI Staining): MCF7 cells from the groups were harvested and re-suspended in 1× binding buffer. They were then stained with AnnexinV-APC and Propidium Iodide (PI) as per kit instructions (BD Biosciences, San Jose, CA, USA). After a 15 min incubation at room temperature in the dark, the stained cells were analyzed by flow cytometry to quantify the apoptosis. This experiment was designed to determine if the pro-proliferative effect of *DHCR24* knockdown was accompanied by a concomitant reduction in apoptosis.

Cell cycle: MCF7 cells were harvested and fixed in ice-cold 70% ethanol for overnight at 4 °C. Fixed cells were centrifuged (2000 rpm/min, 3 min), washed with PBS, and resuspended in PBS with RNase A (100 µg/mL) and PI (50 µg/mL) (Sigma-Aldrich, St. Louis, MO, USA). After a 30 min incubation for room temperature at 37 °C, cell cycle distribution was assessed by flow cytometry measuring PI fluorescence. Flow cytometric analysis was conducted to investigate if *DHCR24* influences cell cycle progression, providing a potential mechanism for its effect on proliferation.

Cell migration (transwell assay): Serum-starved (4–6 h) MCF7 cells in logarithmic phase were harvested, washed and resuspended. Cell density was adjusted to 2 × 10^5^/mL. 100 µL cell suspension was seeded into the upper chamber of a Transwell insert (Corning Life Sciences, Corning, NY, USA), and the lower chamber was filled with medium containing 10% FBS. After 15 h of incubation at 37 °C, non-migrated cells were removed and migrated cells were fixed with 4% PFA (Electron Microscopy Sciences, Hatfield, PA, USA), stained with 0.5% crystal violet (Sigma-Aldrich, St. Louis, MO, USA), washed with PBS, air-dried, and imaged. Migrated cells were counted microscopically. This functional assay was crucial to assess the role of *DHCR24* in regulating cell motility, a key step in the metastatic cascade.

Cell invasion (Matrigel-coated transwell assay): Matrigel matrix (Corning Life Sciences, Corning, NY, USA) was diluted 1:10 in cold serum-free medium and pre-coated onto Transwell inserts (100 µL/chamber), followed by a 4 h polymerization at 37 °C. The upper chamber’s mixture was replaced with serum-free medium. ell density was adjusted to 6 × 10^5^/mL, and the remaining steps were performed as in the Transwell assay. To build upon the migration results and specifically evaluate the ability of cells to degrade the extracellular matrix and invade—a more aggressive malignant phenotype—we performed this assay.

For quantification, five random fields per insert were photographed under a light microscope, and the number of cells was counted using ImageJ software v1.54g (Bethesda, MD, USA). To control for any potential effect of differential cell proliferation or viability on seeding density, the counted cell numbers were normalized to the relative cell viability assessed by a parallel CCK-8 assay at the time of seeding. Results are expressed as the mean ± SD of the normalized cell count per field from three independent experiments. The results from these functional assays provided the critical experimental evidence linking *DHCR24* knockdown to enhanced aggressive phenotypes in vitro, a finding we then sought to reconcile with its patient-level prognostic association through subsequent mechanistic investigations.

### 2.4. Molecular Mechanism Investigation

Following the functional validation, we employed a series of bioinformatic analyses to elucidate the potential molecular mechanisms through which *DHCR24* influences breast cancer biology.

#### 2.4.1. Differential Expression Analysis

Samples in TCGA-BRCA were categorized into low-(0~50% quantile, reference) and high-expression (50~100% quantile) groups using *DHCR24* expression median. With |LogFC| > 1 and p.adj < 0.05, differentially expressed genes were found by DESeq2 package in R (version 4.2.1), and visualized via ggplot2 package. This analysis aimed to identify the global transcriptomic changes induced by high *DHCR24* expression, offering insights into its downstream regulatory networks.

#### 2.4.2. Enrichment Analyses

The differentially expressed genes obtained above were converted using the org.Hs.eg.db package. And enrichment analyses were performed via the cluster Profiler package, covering GO enrichment, KEGG pathway, and gene set enrichment analysis (GSEA). The ggplot2 package was used for visualizing the enrichment results. Pathway enrichment analysis was performed to interpret the biological significance of the *DHCR24*-associated gene signature, pinpointing the key biological processes and pathways it modulates.

#### 2.4.3. Protein–Protein Interaction (PPI) Network Construction

DHCR24-interacting and related proteins were identified with the STRING database (https://string-db.org, accessed on 15 June 2023) [28]. PPI networks were visualized and analyzed through Cytoscape v3.9.1 [29]. Hub genes were identified via CytoHubba based on maximal clique centrality (MCC) scores. Constructing the PPI network allowed us to identify the core functional modules and hub genes that physically or functionally interact with *DHCR24*, providing a systems-level view of its role.

#### 2.4.4. EMT-Associated Gene Analysis

EMT-related genes were retrieved from EMTome (http://emtome.org, accessed on 11 April 2024) [30] to check the link between *DHCR24* and BC EMT. Given the observed changes in migration and invasion upon *DHCR24* knockdown, we specifically interrogated the overlap between our DEGs and a canonical EMT signature to determine if *DHCR24* promotes a partial EMT.

### 2.5. Statistical Analysis

In this study, RNA-seq data processing, differential expression analysis, enrichment analyses, and data visualization were carried out using R (version 4.2.1). For the immunohistochemistry and functional assay data, SPSS (version 26.0) was utilized. When comparing four-grid tables, chi-squared tests were applied for those with consistent numbers, while the exact probability method was used for inconsistent ones. One-way ANOVA followed by Tukey’s post hoc test was employed for the functional assays. A *p*-value < 0.05 was considered statistically significant. This multi-faceted statistical approach, tailored to each specific data type (omics vs. experimental), was integral to ensuring the robustness and reliability of conclusions drawn across our integrated multi-omics strategy.

### 2.6. AI Assistance Disclosure

During the preparation of this work, the authors used Deepseek-v3.1 only to improve readability and language. After using this tool, the authors reviewed and edited the content as needed and take full responsibility for the content of the publication.

## 3. Results

### 3.1. Genetic and Clinical Evidence Links Cholesterol Metabolism and DHCR24 to BC

#### 3.1.1. Causal Link Between Cholesterol and BC Risk

**Figure 1:**
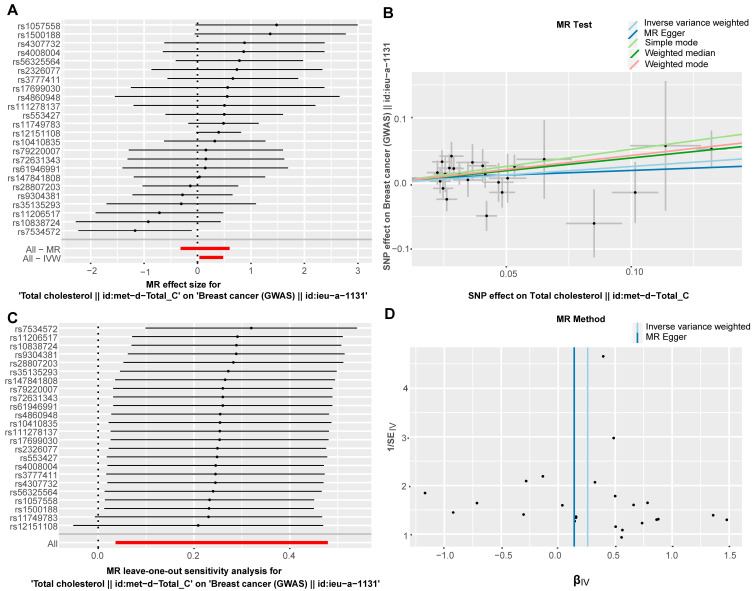
**Elevated total cholesterol has a causal effect on BC risk.** (**A**): Forest Plot of the inverse variance weighted (IVW) MR analysis; (**B**): Scatter Plot of MR analysis; (**C**): Leave-One-Out sensitivity analysis; (**D**): Funnel Plot for assessing pleiotropy. MR, Mendelian randomization. Short red lines (**A**) denote the overall causal estimates from MR-Egger (All-MR) and IVW (All-IVW) using all SNPs. The long red line (**C**) shows the IVW estimate and 95% CI after sequentially omitting each SNP (leave-one-out sensitivity analysis).

This study used a two-sample mendelian randomization (MR) to explore the causal relationship between total cholesterol levels and BC risk. We selected 24 independent single-nucleotide polymorphisms (SNPs) strongly associated with total cholesterol (*p* < 5 × 10^−8^, linkage disequilibrium r^2^ < 0.001) as instrumental variables. Using the inverse-variance weighted (IVW) as primary method, we found that each genetically determined increase in total cholesterol was linked to a higher BC risk (OR = 1.294, 95% CI: 1.037–1.615, *p* = 0.0225, IVW method). Sensitivity analyses confirmed the robustness of the results, which revealed no evidence of significant horizontal pleiotropy (MR-Egger intercept test *p* = 0.572) or substantial heterogeneity (Cochran’s Q *p* = 0.396), and excluding each instrumental variable in turn did not significantly change the results. These results provide genetic evidence supporting total cholesterol as a causal, modifiable risk factor for BC, which establishes a foundational rationale for investigating downstream cholesterol metabolic pathways in disease pathogenesis (Figure 1).

#### 3.1.2. Multi-Omics Screening Identifies *DHCR24* as a Key Mediator in BC

**Figure 2:**
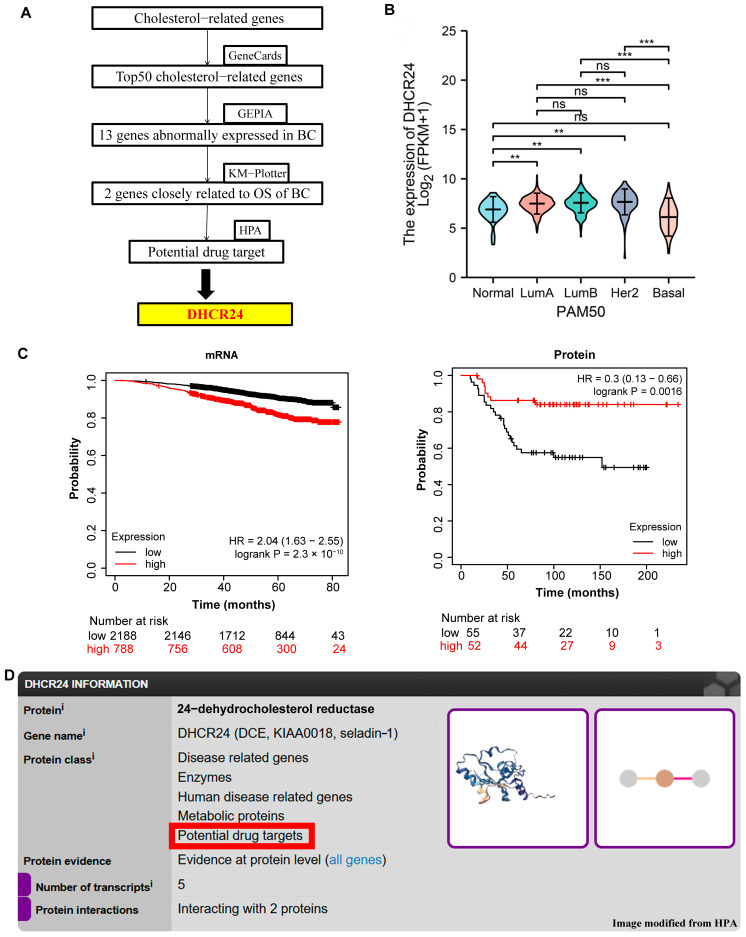
**Multi-omics screening identifies *DHCR24* as a key mediator linking cholesterol metabolism to BC**. (**A**): Flowchart of the integrative multi-omics screening strategy; (**B**): *DHCR24* mRNA expression across PAM50 molecular subtypes of BC; (**C**): Association between overall survival and *DHCR24* in BC in relation to mRNA (**left**) and protein (**right**) levels, analyzed by KM-Plotter; (**D**): Screenshot from the HPA database classifying *DHCR24* within the ‘potential drug target’. ***: *p* < 0.001; **: *p* < 0.01; ns: *p* > 0.05.

To identify cholesterol metabolism genes involved in BC development, we employed a multi-omics screening strategy, integrating data from the GeneCards database and validated expression patterns using GEPIA. Among the 50 top-ranking candidates, 13 genes showed differential expressed in BC (Figure 2A, and their expression patterns across multiple cohorts are detailed in Supplementary Figure S1A). *DHCR24* was upregulated in luminal/HER2+ subtypes but unchanged in basal-like BC (Figure 2B). Survival analysis using KM-Plotter indicated that *DHCR24* and *NCEH1* were correlated with overall survival at both mRNA and protein levels (Figure 2C, Supplementary Figure S1B). Intriguingly, we observed a dissociation between molecular layers: high *DHCR24* mRNA expression was associated with poor overall survival (HR = 2.04, *p* = 2.3 × 10^−10^), whereas high protein expression was linked to better survival (HR = 0.3, *p* = 0.0016). This discrepancy highlights the complex post-transcriptional regulation of *DHCR24* and suggests that its mRNA level may be a more immediate reflection of its biological activity in these contexts. Importantly, analysis of the HPA database classified *DHCR24* within the ‘potential drug target’ (Figure 2D). This multi-faceted profiling—encompassing differential expression, strong subtype-specific prognostic power, and druggability annotation—collectively motivated the selection of *DHCR24* for in-depth functional and mechanistic investigation.

**Figure 3:**
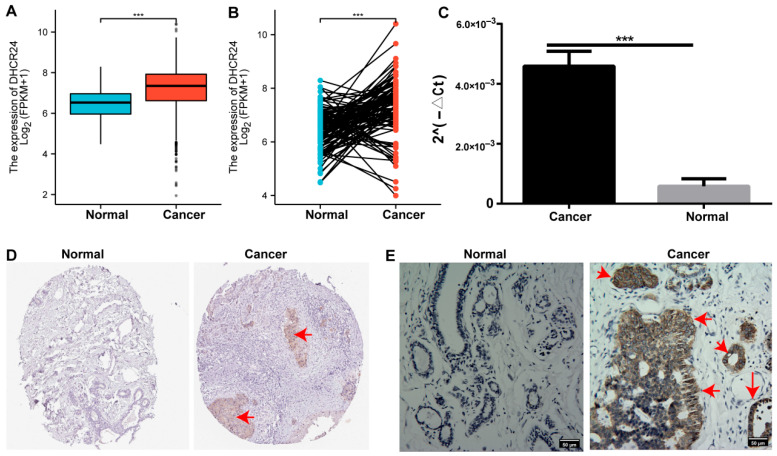
**DHCR24 is dysregulated in BC tissues.** (**A**,**B**): *DHCR24* mRNA expression levels in (**A**) unpaired and (**B**) paired BC and normal tissues from the TCGA and GTEx cohorts; (**C**): Experimental verification in our cohort by RT-qPCR (*n* = 3); (**D**,**E**): *DHCR24* protein expression in (**D**) BC and normal tissues from the HPA database and (**E**) validated by immunohistochemistry (IHC) in our cohort. Red arrows indicate representative areas demonstrating significantly high *DHCR24* expression in the BC when compared to the normal tissue (representative images). BC, breast cancer; ***: *p* < 0.001. Scale bars, 50 μm.

Notably, the oncogenic role of *DHCR24* appears to extend beyond BC, as it was found to be significantly upregulated in a wide panoply of human malignancies (Figure S2A,B). Specifically, in BC, *DHCR24* levels were significantly increased compared to normal tissues, as observed at both mRNA (Figure 3A–C) and protein levels (Figure 3D,E).

Immunohistochemical validation (Table 1) confirmed several key associations: PR+ (*p* = 0.028) and HER2+ (*p* = 0.022) statuses were correlated with increased *DHCR24* expression, whereas TNBC status showed an inverse correlation (*p* = 0.019). No significant association was observed with ER status (*p* = 0.758). A comprehensive analysis of clinical correlations further reinforced that high *DHCR24* expression is a feature of Luminal and HER2+ subtypes, and this association was independent of menopausal status or race (Figure S3A–F). Additionally, *DHCR24* expression differed significantly between patients over 60 years old and those under 60 (Figure S3D). Having established *DHCR24* as a clinically relevant factor with subtype-specific expression and paradoxical prognostic associations, we next sought to functionally characterize its role in oncogenic phenotypes in vitro.

### 3.2. DHCR24 Knockdown Promotes Oncogenic Phenotypes in BC Cells

#### 3.2.1. Expression Profiling Informs Experimental Selection

**Figure 4:**
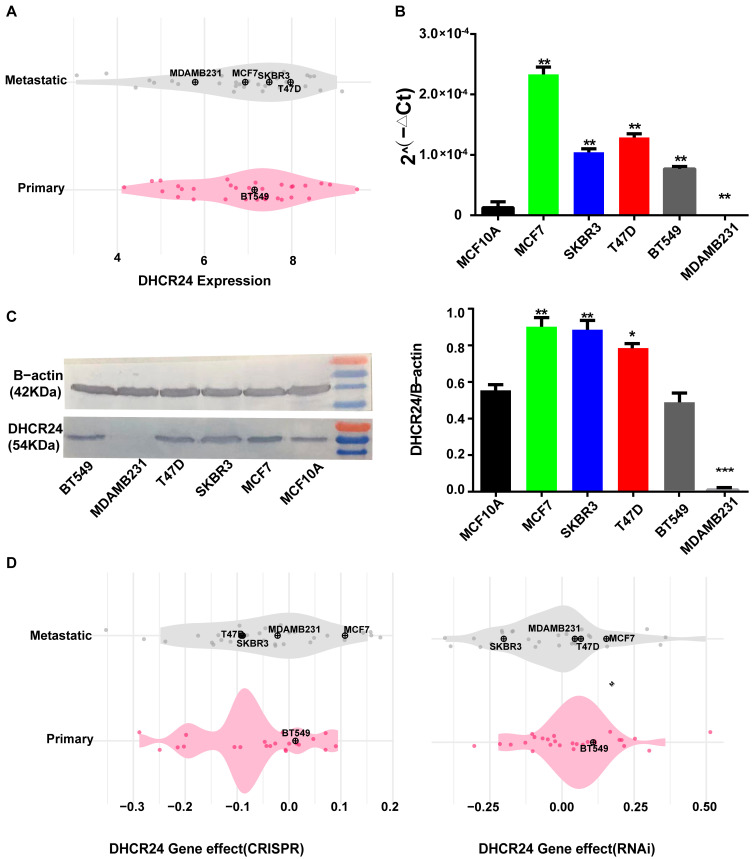
**DHCR24 is essential for BC cell fitness**. (**A**): *DHCR24* mRNA expression across a panel of BC cell lines; (**B**,**C**): Validation of *DHCR24* (**B**) mRNA and (**C**) protein expression in selected cell lines; (**D**): Dependency scores from the DepMap database show that BC cell viability is significantly reduced upon (**left**) CRISPR knockout or (**right**) RNAi knockdown of *DHCR24*, indicating it is a dependency gene. ***: *p* < 0.001; **: *p* < 0.01; *: *p* < 0.05.

We analyzed *DHCR24* levels across various BC cell lines. Results showed that *DHCR24* levels varied among cancer types, but remained relatively stable in BC (Figure 4A). We then focused on the MCF7, MDAMB231, BT549, SKBR3, and T47D cell lines. As shown in Figure 4A, *DHCR24* expression level was highest in T47D, followed by SKBR3 and BT549, then MCF7, and lowest in MDAMB231. Compared to the control MCF10A cell line, *DHCR24* levels were markedly higher in MCF7, T47D, and SKBR3 cells, while MDAMB231 showed lower expression (Figure 4B,C, original figures see File S1). Using the Depmap Portal database, we predicted the effects of altering *DHCR24* expression in these BC cells. The findings suggested that changing *DHCR24* levels via RNAi would likely have a pronounced effect on MCF7, BT549 and T47D cells. On the other hand, CRISPR-mediated perturbation would mainly affect MCF7 cells (Figure 4D). Based on these results, which indicated a predicted strong functional dependency in MCF7 cells, we selected this luminal model for subsequent functional validation to dissect the cell-autonomous effects of *DHCR24*.

#### 3.2.2. *DHCR24* Knockdown Promotes Malignant Phenotypes in MCF7 Cells

**Figure 5:**
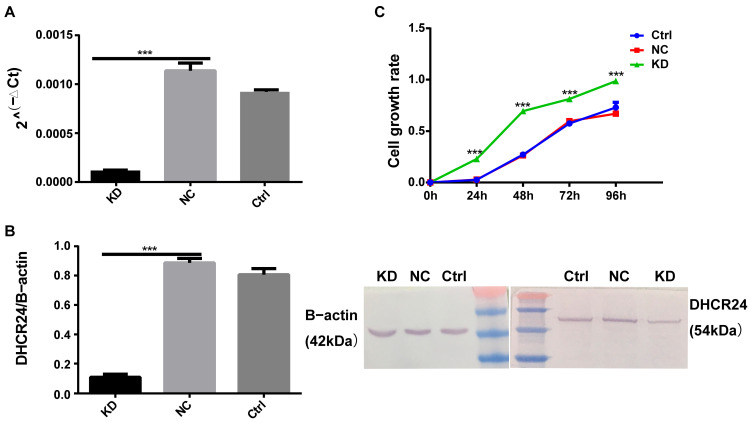
**DHCR24 knockdown promotes proliferation of MCF7 cells**. (**A**,**B**): Efficiency of *DHCR24* knockdown in MCF7 cells confirmed at the (**A**) mRNA and (**B**) protein levels; (**C**): CCK-8 proliferation assay demonstrating that *DHCR24* KD significantly increased cell proliferation compared to the NC group. Data are mean ± SD (*n* = 3). ***: *p* < 0.001. Ctrl, untreated control; NC, negative control; KD, Knockdown. original figures see File S1.

To functionally validate the role of *DHCR24* suggested by our bioinformatic analyses, we performed lentivirus-mediated knockdown in luminal MCF7 cells. Knockdown efficiency was confirmed by a significant reduction in both *DHCR24* mRNA and protein levels(Figure 5A,B). Strikingly, and contrary to a simplistic oncogene model, CCK-8 assays showed a significant increase in cell proliferation following *DHCR24* knockdown compared to the control (Figure 5C). This pro-proliferative effect was consistent with the negative genetic dependency scores (CRISPR/RNAi) for *DHCR24* in BC cell lines from the DepMap database (Figure 4D), suggesting that in the specific context of MCF7 cells, *DHCR24* may function to restrain rather than drive cell-autonomous growth.

**Figure 6:**
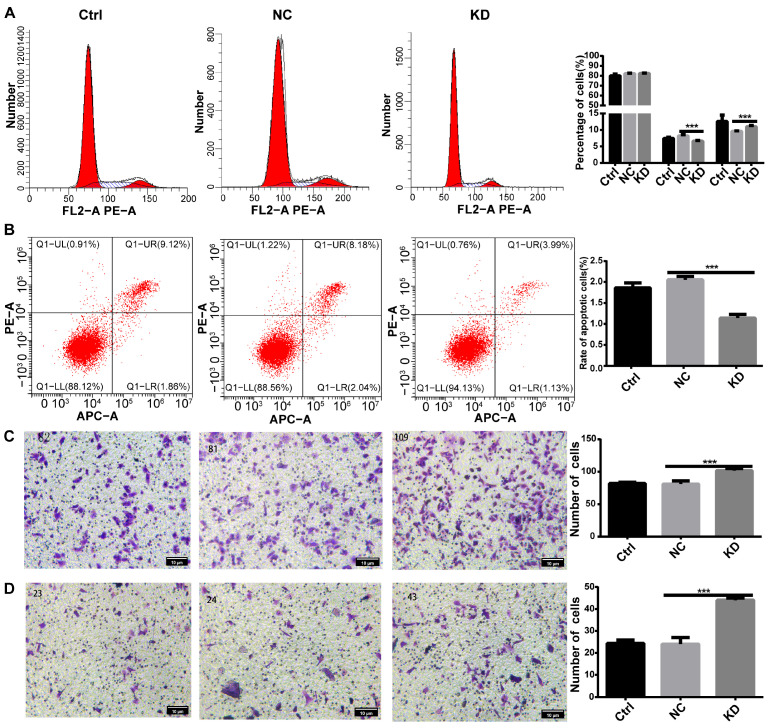
**DHCR24 knockdown enhances malignant phenotypes in MCF7 cells.** (**A**): Flow cytometry analysis showing that *DHCR24* knockdown alters cell cycle distribution; (**B**): Apoptosis assay reveals that knockdown reduces apoptosis; (**C**,**D**): Transwell assays demonstrate that *DHCR24* knockdown significantly enhances the (**C**) migratory and (**D**) invasive capabilities of MCF7 cells (representative images). Data are mean ± SD (*n* = 3). ***: *p* < 0.001. Scale bars, 10 μm.

We further investigated the impact of *DHCR24* knockdown on other core oncogenic phenotypes. Cell cycle analysis by flow cytometry showed a significant increase in the proportion of cells in S phase upon *DHCR24* knockdown (Figure 6A), indicating an accelerated cell cycle progression that mechanistically explains the observed proliferation boost. Corroborating this, apoptosis was significantly reduced in the knockdown group (Figure 6B). Moreover, *DHCR24* knockdown conferred enhanced invasive and migratory capacities in Transwell assays (Figure 6C,D). Taken together, these functional assays reveal a clear and counterintuitive phenotype: genetic depletion of *DHCR24* enhances the malignant potential of MCF7 cells by promoting proliferation, reducing apoptosis, and increasing invasiveness. This cell-autonomous gain-of-function upon loss-of-*DHCR24* stands in apparent contradiction to its association with poor patient prognosis, prompting the hypothesis that its primary oncogenic role in vivo may be non-cell-autonomous, potentially mediated through the tumor microenvironment.

#### 3.2.3. *DHCR24* Knockdown Induces a Partial EMT Phenotype

**Figure 7:**
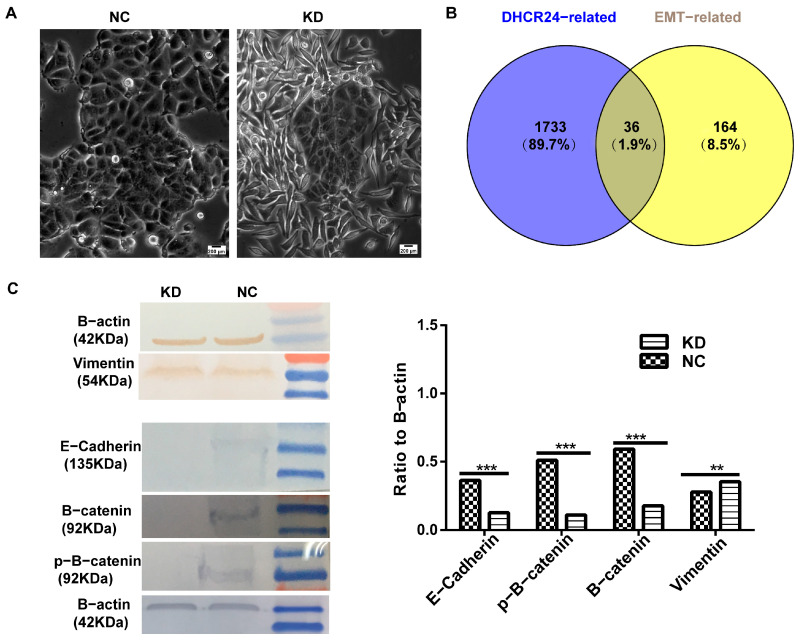
**DHCR24 knockdown induces a partial epithelial–mesenchymal transition (EMT).** (**A**): Bright-field images showing a morphological shift towards a more spindle-like, mesenchymal phenotype upon *DHCR24* knockdown (representative images); (**B**): Venn diagram identifying a significant overlap between genes differentially expressed after *DHCR24* knockdown and a canonical EMT gene set; (**C**): Western blot analysis confirms the downregulation of the epithelial marker *E-cadherin* and upregulation of the mesenchymal markers *Vimentin* following *DHCR24* knockdown. For optimal detection, the analysis of *E-cadherin* and *Vimentin* was performed in two independent experiments, each containing its own *β-actin* loading control, original figures see File S1. Scale bar, 200 μm (applies to panel (**A**)). ***: *p* < 0.001; **: *p* < 0.01.

Given the observed increase in invasive capacity induced by *DHCR24* knockdown (Figure 6C,D), coupled with the observation that MCF7 cells acquired a more mesenchymal morphology (Figure 7A), we investigated its potential role in regulating epithelial–mesenchymal transition (EMT). Bioinformatic interrogation of the EMTome database identified 36 overlapping genes (1.9%) between EMT-related genes and DEGs from tumors with high- and low-*DHCR24* expression (Figure 7B). Critically, to obtain direct experimental validation of this connection, we analyzed the expression of core EMT marker proteins. Western blot analysis yielded clear and compelling evidence: *DHCR24* knockdown potently decreased the expression of key epithelial markers (*E-cadherin*, *β-catenin*, and *p-β-catenin*) while concurrently increasing the expression of the mesenchymal marker *Vimentin* (Figure 7C). This coherent shift in the protein expression landscape demonstrates conclusively that *DHCR24* depletion drives a partial EMT program in MCF7 cells. This molecular transition provides the definitive mechanistic explanation for the enhanced invasive and migratory phenotypes observed in Figure 6C,D, functionally linking the loss of *DHCR24* to increased cellular plasticity, a hallmark of aggressive disease.

### 3.3. Multi-Omics Analyses Implicate DHCR24 in Tumor Microenvironment Remodeling and Transcriptional Networks

To explore the mechanisms underlying the clinical associations of *DHCR24*, we performed integrated multi-omics analyses. Enrichment analyses (GSEA, GO and KEGG) of *DHCR24*-associated genes and hub genes from a protein–protein interaction (PPI) network revealed significant enrichment in processes related to immune response and cholesterol metabolic, Specifically, GSEA highlighted the significant relationship of BCR antigen activation and complement cascade pathways in high-*DHCR24* tumors, while KEGG analysis revealed marked enrichment in neuroactive ligand–receptor interactions and cytokine–cytokine receptor interactions. This multi-omic signature strongly implicates *DHCR24* at the critical intersection of cellular metabolism and immune regulation, providing a plausible mechanistic bridge between the enzyme and microenvironmental modulation.

**Figure 8:**
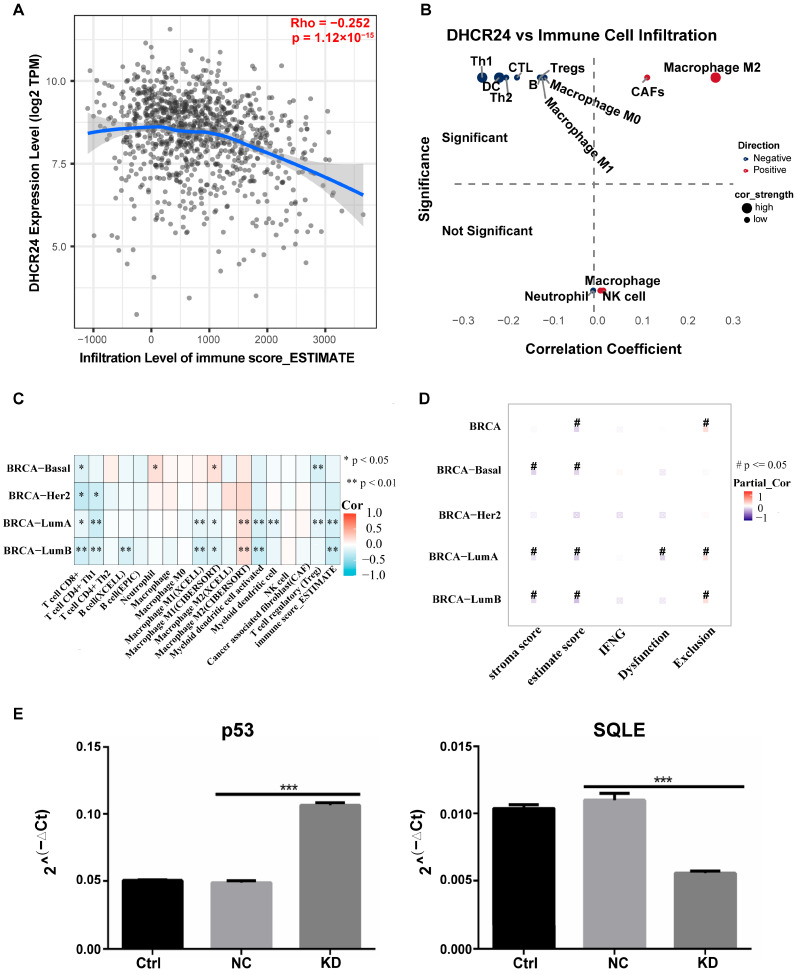
**DHCR24 expression is associated with an immunosuppressive tumor microenvironment and modulates key transcriptional networks.** (**A**): *DHCR24* mRNA expression negatively correlates with immune score in the TCGA-BRCA cohort; (**B**): Correlation analysis between *DHCR24* expression and the abundance of various tumor-infiltrating immune cells; (**C**): Proposed model: *DHCR24* contributes to an immunosuppressive microenvironment across BC subtypes; (**D**,**E**): The mRNA expression levels of (**D**) *TP53* and (**E**) *SQLE* was significantly upregulated and downregulated upon *DHCR24* knockdown in MCF7 cells, respectively. Data are mean ± SD (*n* = 3). *: *p* < 0.05; **: *p* < 0.01; ***: *p* < 0.001. #: *p* <= 0.05.

Our next study comprehensively evaluated the relationship between *DHCR24* expression and the TME landscape. Using the ESTIMATE algorithm, we observed a significant negative correlation between *DHCR24* mRNA levels and the Immune Score (Figure 8A). This key finding indicates that higher *DHCR24* expression is associated with a globally suppressed or “cold” immune landscape within tumors, aligning with its poor prognostic association. Further analysis of specific immune cell subsets revealed a distinct pattern: *DHCR24* expression was negatively correlated with the abundance of anti-tumor immune cells (e.g., CD8+ T cells, CD4+ Th1 cells) and positively correlated with pro-tumorigenic components (e.g., M2 macrophages, cancer-associated fibroblasts) (Figure 8B). Importantly, this immunomodulatory role of *DHCR24* was not uniform. Analysis across molecular subtypes revealed clear variation in the strength and nature of these associations (Figure 8C), with the most pronounced and consistent correlations observed in the Luminal A and Luminal B subtypes. For instance, in Luminal A, *DHCR24* levels strongly correlated with both immune exclusion and dysfunction signatures, underscoring a multi-faceted role in immune evasion within this context. This suggests that *DHCR24*’s impact on the TME is context-dependent, with potentially greater relevance in the Luminal and HER2+ subtypes where it is highly expressed. This subtype-specific immunomodulatory pattern provides a compelling, non-cell-autonomous mechanism that can reconcile the pro-tumorigenic role of high *DHCR24* in patients with the paradoxical cell-autonomous phenotypes observed upon its knockdown in vitro.

To molecularly define potential mechanisms by which *DHCR24* influences the tumor immune microenvironment, we constructed a protein–protein interaction (PPI) network centered on *DHCR24*. Network analysis using the Maximal Clique Centrality (MCC) method identified several high-confidence interactors, with the tumor suppressor *TP53* and the rate-limiting cholesterol synthesis enzyme *SQLE* emerging as the top-tier hub genes (Supplementary Figure S5C), thereby nominating two pivotal, functionally distinct pathways through which *DHCR24* may exert its effects. Strikingly, experimental validation in MCF7 cells showed that *DHCR24* knockdown led to a significant upregulation of *TP53* and downregulation of *SQLE* at the mRNA level (Figure 8E). This reciprocal regulation suggests a sophisticated regulatory circuit where *DHCR24* potentially suppresses *TP53* activity and sustains cholesterol biosynthesis, mechanisms that are both known to influence immune cell function and tumor surveillance. Collectively, these data support a model wherein *DHCR24*, potentially via modulating the *p53* pathway and cholesterol synthesis (through *SQLE*), contributes to shaping an immunosuppressive TME. This dual-axis mechanism provides a compelling molecular explanation for the observed correlation between high *DHCR24*, a “cold” immune microenvironment, and poor patient outcome, effectively bridging our cellular findings with the clinical phenotype.

## 4. Discussion

Breast cancer (BC) is a prevalent malignancy characterized by metabolic reprogramming [3,31]. We move beyond association by demonstrating a causal link between cholesterol and BC risk via Mendelian randomization [32] and, through unbiased screening, nominate the biosynthetic enzyme *DHCR24* as a central mediator. Our data compellingly argue that *DHCR24* is not a mere metabolic housekeeper but a pleiotropic regulator of tumor progression, primarily by sculpting an immunosuppressive tumor microenvironment (TME), reconciling the apparent paradox between its pro-tumor role in patients and cell-autonomous effects in vitro.

A cornerstone of our findings is the robust association between high *DHCR24* expression and an immunosuppressive TME. Our integrated analyses using both ESTIMATE [33] and TIDE [34] algorithms consistently demonstrate that elevated *DHCR24* levels correlate with a globally “cold” immune landscape. This is characterized not only by a depleted overall Immune Score but also by a significantly enhanced Immune Exclusion score, suggesting an active mechanism by which *DHCR24*-expressing tumors resist lymphocyte infiltration. At the cellular level, this niche is further defined by depleted cytotoxic T cells and enriched pro-tumorigenic components like M2 macrophages.

Building on the established role of cholesterol metabolites in shaping immune functions [35,36,37,38], our bioinformatic analyses suggest a dual-axis mechanistic model: *DHCR24* may foster this niche by potentially modulating the *p53* tumor suppressor pathway while concurrently driving cholesterol synthesis via *SQLE*. This model, supported by our preliminary validation, posits that *DHCR24* tunes the metabolic-immune dialog within the TME, potentially influencing both the composition and functional state of immune cells, as indicated by its association with T-cell dysfunction signatures in specific subtypes like Luminal A. However, these specific mechanistic interactions require direct experimental confirmation in future studies.

The most intriguing paradox emerged from functional validation: *DHCR24* knockdown in luminal MCF7 cells enhanced aggressive, cell-autonomous phenotypes—proliferation, invasion, and EMT. This apparent contradiction with its pro-tumorigenic organismal role underscores a critical concept in cancer metabolism: the distinction between cell-autonomous and non-cell-autonomous functions [39]. We propose that the cell-intrinsic effects represent a context-specific adaptation. The concurrent upregulation of *TP53* upon knockdown offers a crucial clue; *p53*’s context-dependent role in cell fate decisions may underlie this unexpected invasiveness, though the precise effector mechanisms remain to be fully elucidated and may involve *p53*-independent pathways [40,41].

We further observed a dissociation between *DHCR24*’s prognostic value at mRNA versus protein levels. While high mRNA expression consistently predicted poor survival, existing protein-level data showed an opposite trend. This discrepancy underscores complex post-transcriptional regulation, potentially reflecting differential protein stability, translational efficiency, or key methodological differences between assays [34]. Notably, the prognostic power of *DHCR24* mRNA was particularly strong and consistent within specific molecular subtypes, such as Luminal A and B, mirroring the subtype-specific patterns observed in our immune correlation analyses. Given this, the biomarker utility of *DHCR24* appears more robust at the transcriptional level, though further validation of protein-level associations in well-controlled cohorts is warranted.

While our current findings highlight *DHCR24*’s therapeutic potential, we acknowledge that its direct druggability requires further investigation beyond genomic classification. Critically, our findings position *DHCR24* as a compelling target for novel therapeutic strategies. The rationale is strengthened by its dual role as a key metabolic node and immune modulator. Inhibiting cholesterol biosynthesis has shown promise in cancer therapy, with agents like statins being explored in clinical oncology [42,43]. Specifically, targeting downstream enzymes such as *DHCR24* may offer greater therapeutic specificity [44]. Our data provide a mechanistic hypothesis: by inhibiting *DHCR24*, we might simultaneously disrupt tumor-promoting cholesterol synthesis and reverse the immunosuppressive TME—evidenced by its correlation with exclusion and dysfunction signatures—thereby converting “cold” tumors into ones more susceptible to immunotherapy. Therefore, *DHCR24* represents a strategic convergence point where metabolic inhibition could yield concomitant immunostimulatory effects, offering a potential advantage over targeting either pathway independently.

From a personalized medicine perspective, *DHCR24* holds significant value as a stratification biomarker. Assessing its status could help identify patients with a high likelihood of an immunosuppressive TME, who might be prime candidates for combination strategies. Conversely, low *DHCR24* expression in TNBC, associated with features of chemotherapy sensitivity, could guide treatment decisions, moving towards more biology-driven therapy.

The connection of *DHCR24* with metabolic reprogramming carries profound diagnostic significance. Its dysregulation is a potential driver of the metabolic-immune rewiring characteristic of aggressive BC. Therefore, *DHCR24* expression could serve as a functional readout of a tumor’s metabolic-immune state, aiding in molecular subtyping and identifying tumors reliant on this pathway.

## 5. Limitations and Future Directions

Our study has limitations. The cell-intrinsic effects of *DHCR24* were characterized primarily in one cell line (MCF7) and require validation across a broader panel of models representing different BC subtypes. Our TME analyses, while revealing clear subtype-specific associations (e.g., strong immune correlations in Luminal but not Basal subtypes), are primarily correlative and derived from bulk transcriptomics. The precise mechanistic links between the *DHCR24-p53-SQLE* axis and the observed TME alterations remain to be fully established in vivo. Future work must employ immunocompetent in vivo models, 3D culture systems, and patient-derived organoids to validate the non-cell-autonomous mechanisms and therapeutic potential of *DHCR24* modulation.

## 6. Conclusions

In conclusion, our causality-anchored, multi-omics framework identifies *DHCR24* as a key mediator linking cholesterol metabolism to BC progression via immune microenvironment remodeling. We provide a reconciling model: its primary oncogenic role is non-cell-autonomous, orchestrating an immunosuppressive niche characterized by both diminished immune infiltration and altered immune cell function, as captured by complementary bioinformatic platforms. This insight challenges cell-centric views of cancer metabolism and advocates for *DHCR24*’s dual promise as a context-dependent prognostic biomarker for personalized stratification and a novel therapeutic target at the metabolic-immune interface. However, we emphasize that further mechanistic studies and robust validation across models are needed to fully establish *DHCR24*’s role and therapeutic potential in breast cancer.

## Figures and Tables

**Table 1:** Relationships between *DHCR24* levels and clinicopathological parameters in BC.

Groups	Clinicopathological Parameters	Counts	DHCR24		χ^2^	*p*
			Negative	Positive		
Age	≥60	12	4	8	0	1 ^b^
	<60	47	18	29		
ER	Negative	20	8	12	0.095	0.758 ^a^
	Positive	39	14	25		
PR	Negative	32	16	16	4.832	**0.028** ^a^
	Positive	27	6	21		
HER-2	Negative	42	20	22	5.208	**0.022** ^b^
	Positive	17	2	15		
Subtype	TNBC	9	7	2	5.542	**0.019** ^b^
	No-TNBC	50	15	35		

(ER ≥ 25: positive, PR ≥ 25: positive, HER-2 ≥ 3: positive. ^a^: General Chi-square test; ^b^: Continuity correction Chi-square test. bold: *p* < 0.05).

## Data Availability

The data used in this study were obtained from public databases, such as TCGA, GEPIA, IEU Open GWAS database (details shown in the “Materials and methods” section). All datasets are freely available in these public databases without the need for additional requests or permissions.

## Supplementary Materials

The following supporting information can be downloaded at: https://www.mdpi.com/article/10.3390/biology15010040/s1, Figure S1: Cholesterol metabolism genes are dysregulated in breast cancer and show prognostic value; Figure S2: *DHCR24* exhibits a cancer type-specific expression pattern across malignancies; Figure S3: *DHCR24* expression correlates with key clinicopathological features in breast cancer; Figure S4: Multi-omics profiling reveals *DHCR24*’s association with immune and metabolic pathways; Figure S5: Protein–protein interaction network implicates *DHCR24* within functional modules centered on cholesterol and lipid metabolism. File S1: Uncropped Western−blot images.

## Abbreviations

BC	Breast cancer
IHC	Immunohistochemical
PPI	Protein–protein interaction
IVW	Inverse-variance weighted
EMT	Epithelial–Mesenchymal Transition,
MR	Mendelian randomization
TNBC	Triple-negative breast cancer
GWAS	Genome-wide association study
GSEA	Gene set enrichment analysis
LD	Linkage disequilibrium
