# Supplementary material for: Multi-Omics Integration Identifies the Cholesterol Metabolic Enzyme DHCR24 as a Key Driver in Breast Cancer
Source: Biology (Basel). 2025 Dec 25;15(1):40. doi: 10.3390/biology15010040 (PMC12785013; doi:10.3390/biology15010040)
Supplement: Supplementary file 1 [file biology-15-00040-s001.zip › biology-4020702-supplementary/Supplementary figures.pdf]

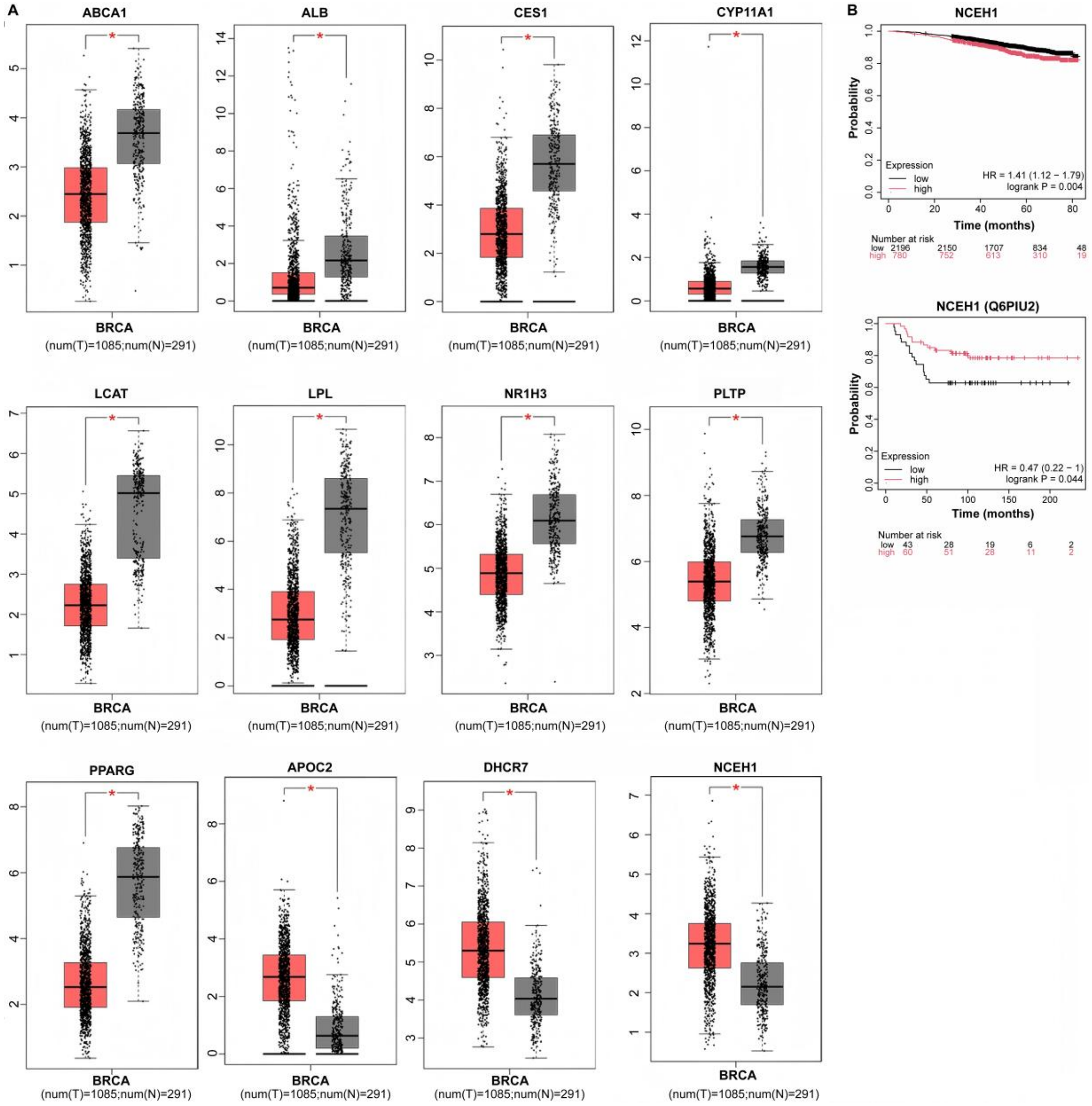

FigureS1 Cholesterol metabolism genes are dysregulated in breast cancer and show prognostic value. A: Boxplot of differentially expressed cholesterol-related genes between breast tumor and adjacent normal tissues; **B:** Kaplan-Meier survival curve showing the prognostic association of NCEH1 mRNA expression with overall survival in BC patients.

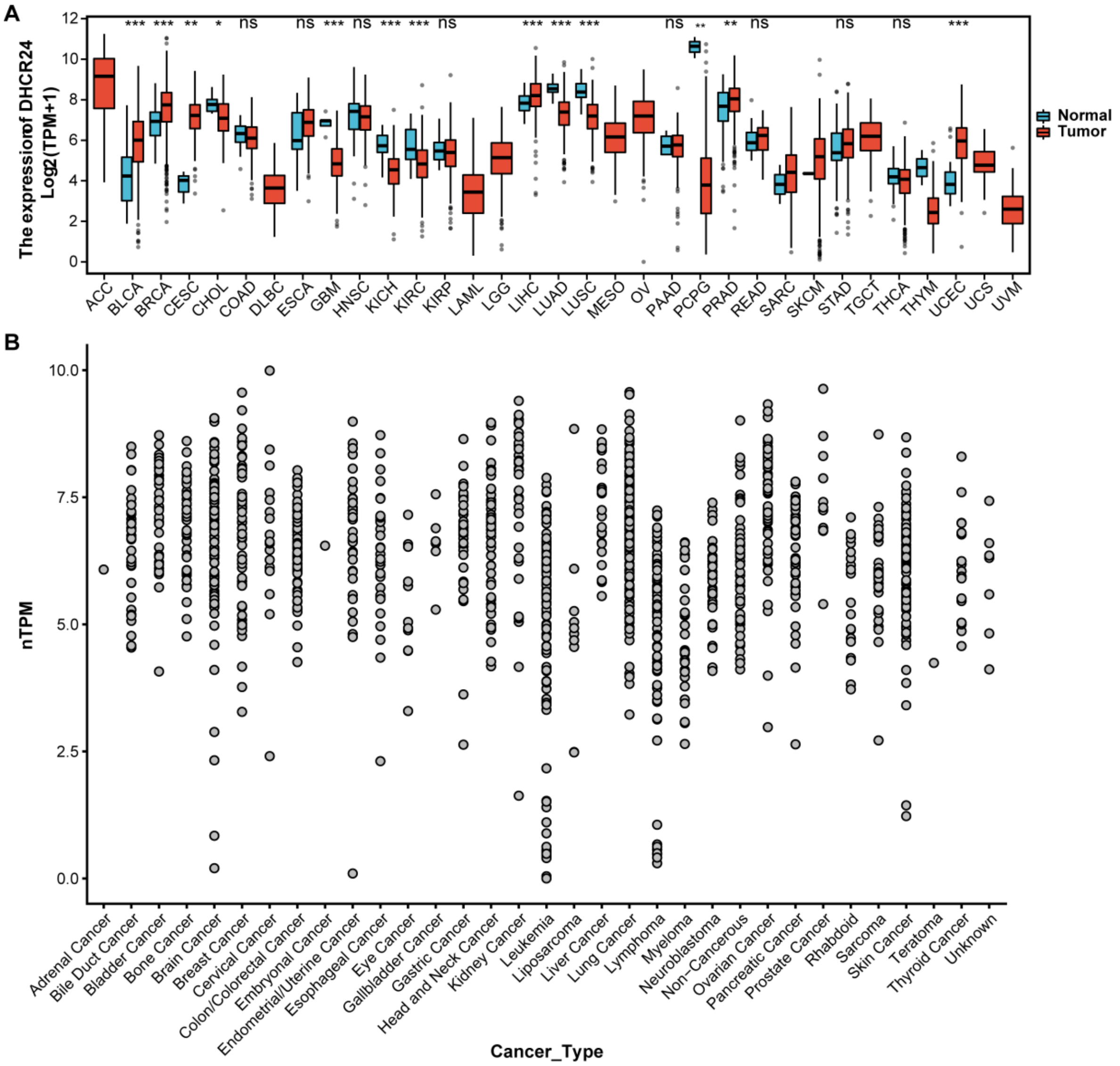

FigureS2 DHCR24 exhibits a cancer type-specific expression pattern across malignancies.
A: DHCR24 mRNA expression levels in tumor versus normal tissues across multiple cancer types from the TCGA and GTEx cohorts; **B:** DHCR24 mRNA expression in a panel of cancer cell lines from the CCLE database.

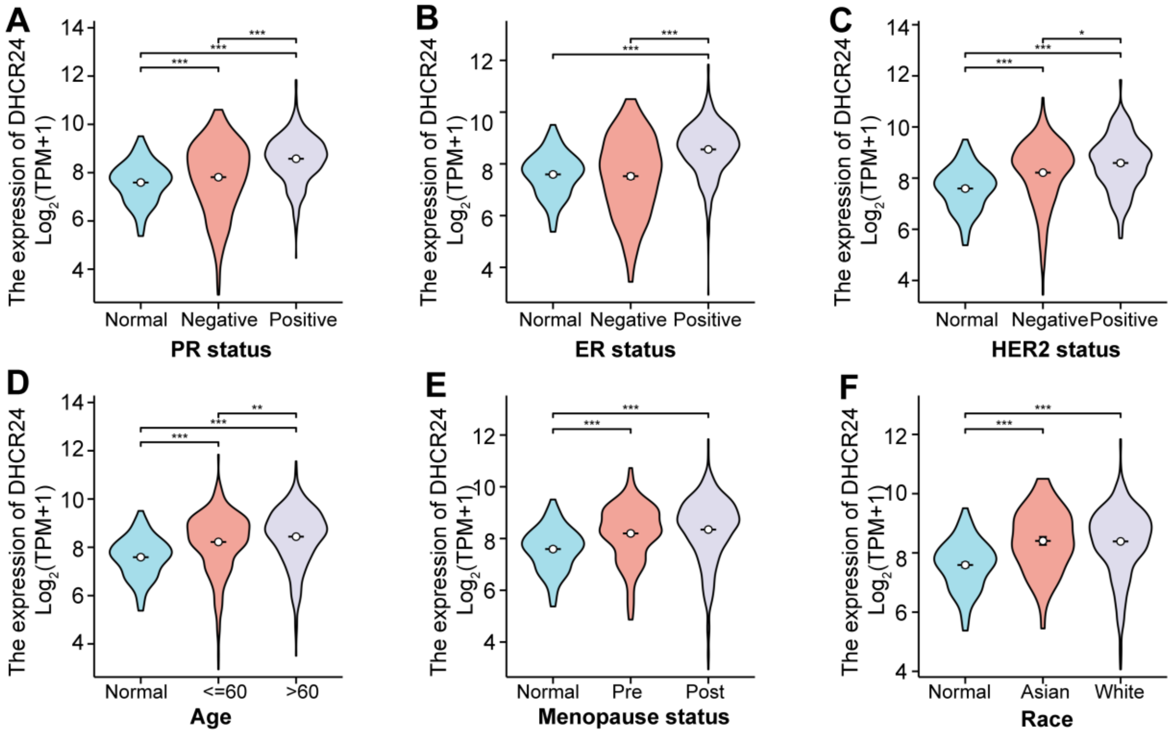

FigureS3 DHCR24 expression correlates with key clinicopathological features in breast cancer. Association of DHCR24 mRNA expression with (A) progesterone receptor (PR) status, (B) estrogen receptor (ER) status, (C) HER2 status, (D) patient age, (E) menopausal status, and (F) race in the TCGA-BRCA cohort. Data are presented as mean ± SD. *: $p < 0.05$; **: $p < 0.01$; ***: $p < 0.001$.

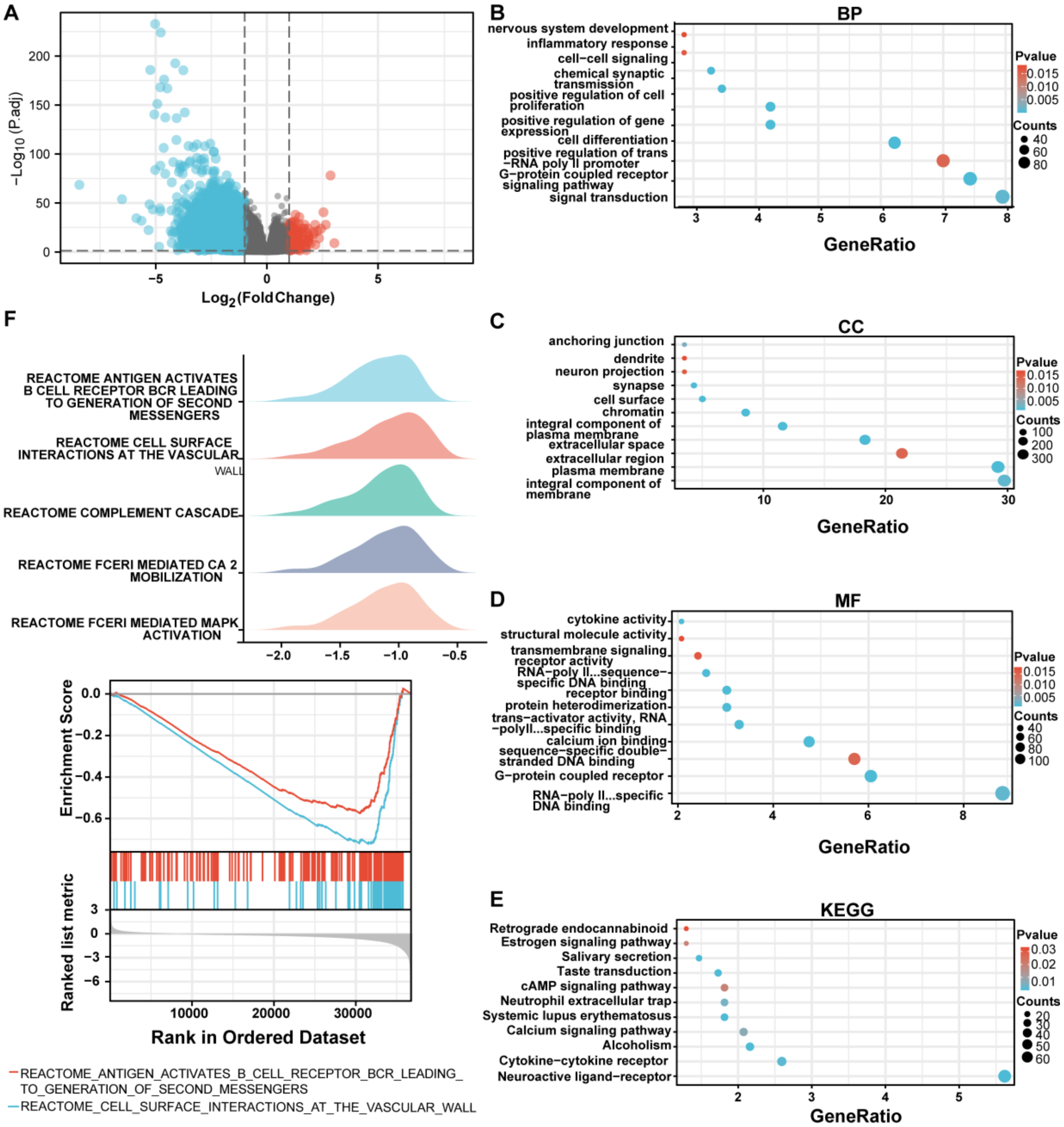

FigureS4 Multi-omics profiling reveals DHCR24's association with immune and metabolic pathways. A: Volcano plot of genes differentially expressed between DHCR24-high and DHCR24-low tumors in the TCGA-BRCA cohort; **B-D:** Gene Ontology (GO) enrichment analysis of these genes in terms of (B) biological processes, (C) cellular components, and (D) molecular functions; **E:** Significantly enriched Kyoto Encyclopedia of Genes and Genomes (KEGG) pathways; **F:**Gene Set Enrichment Analysis (GSEA) results displayed as a ridgeline plot of enriched gene sets (top) and enrichment plots for the top two significant pathways (bottom).

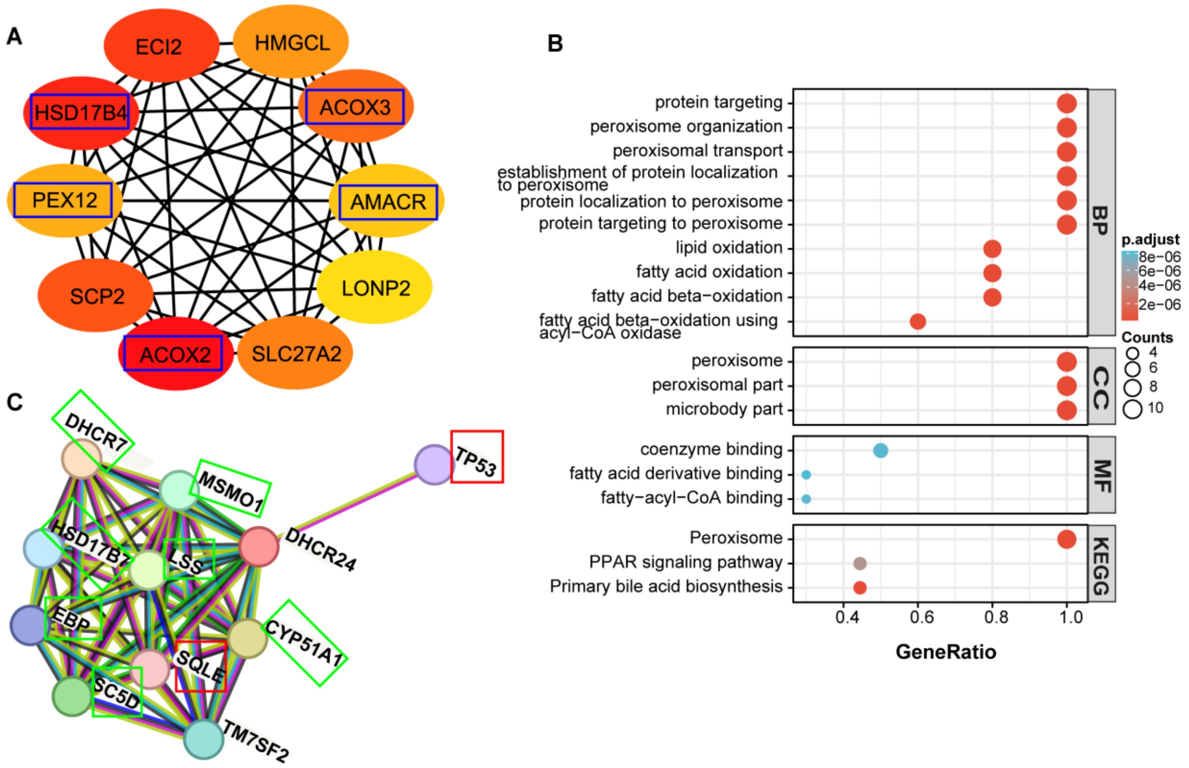

FigureS5 Protein-protein interaction network implicates DHCR24 within functional modules centered on cholesterol and lipid metabolism. **A:** Identification of hub genes from the DHCR24-centric PPI network; **B:** Functional annotation of these hub genes via GO and KEGG analyses; **C:** Direct interaction partners of DHCR24, color-coded by functional module: cholesterol biosynthesis (yellow), lipid transport/metabolism (blue), and peroxisome-related processes (green). Nodes represent proteins; edges represent known interactions.
