# Supplementary figures and images for: Multi-Omics Integration Identifies the Cholesterol Metabolic Enzyme DHCR24 as a Key Driver in Breast Cancer
Source: Biology (Basel). 2025 Dec 25;15(1):40. doi: 10.3390/biology15010040 (PMC12785013; doi:10.3390/biology15010040)

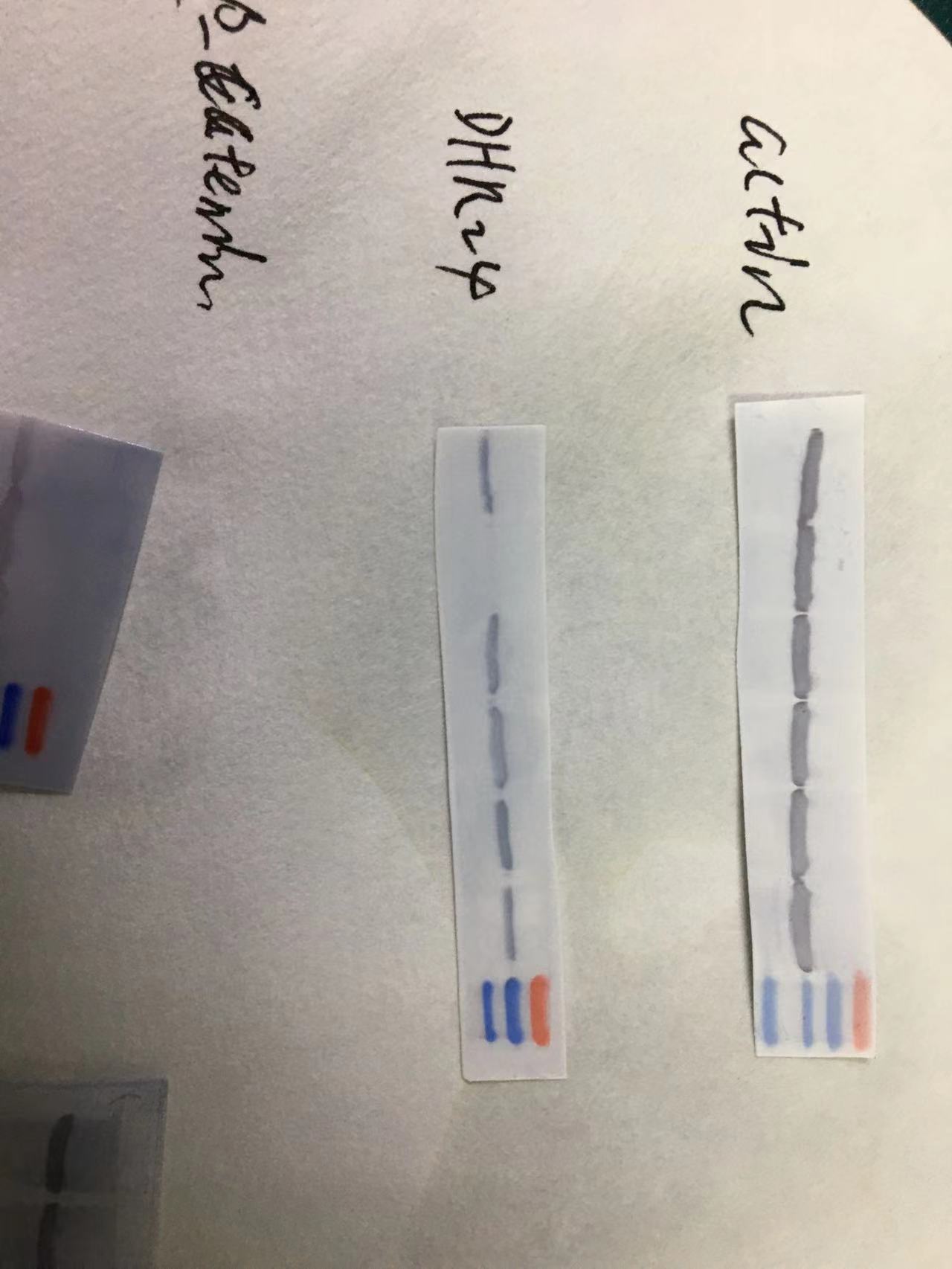

Supplement: Supplementary file 1 [file biology-15-00040-s001.zip › biology-4020702-supplementary/File S1/Figure4C.JPG]

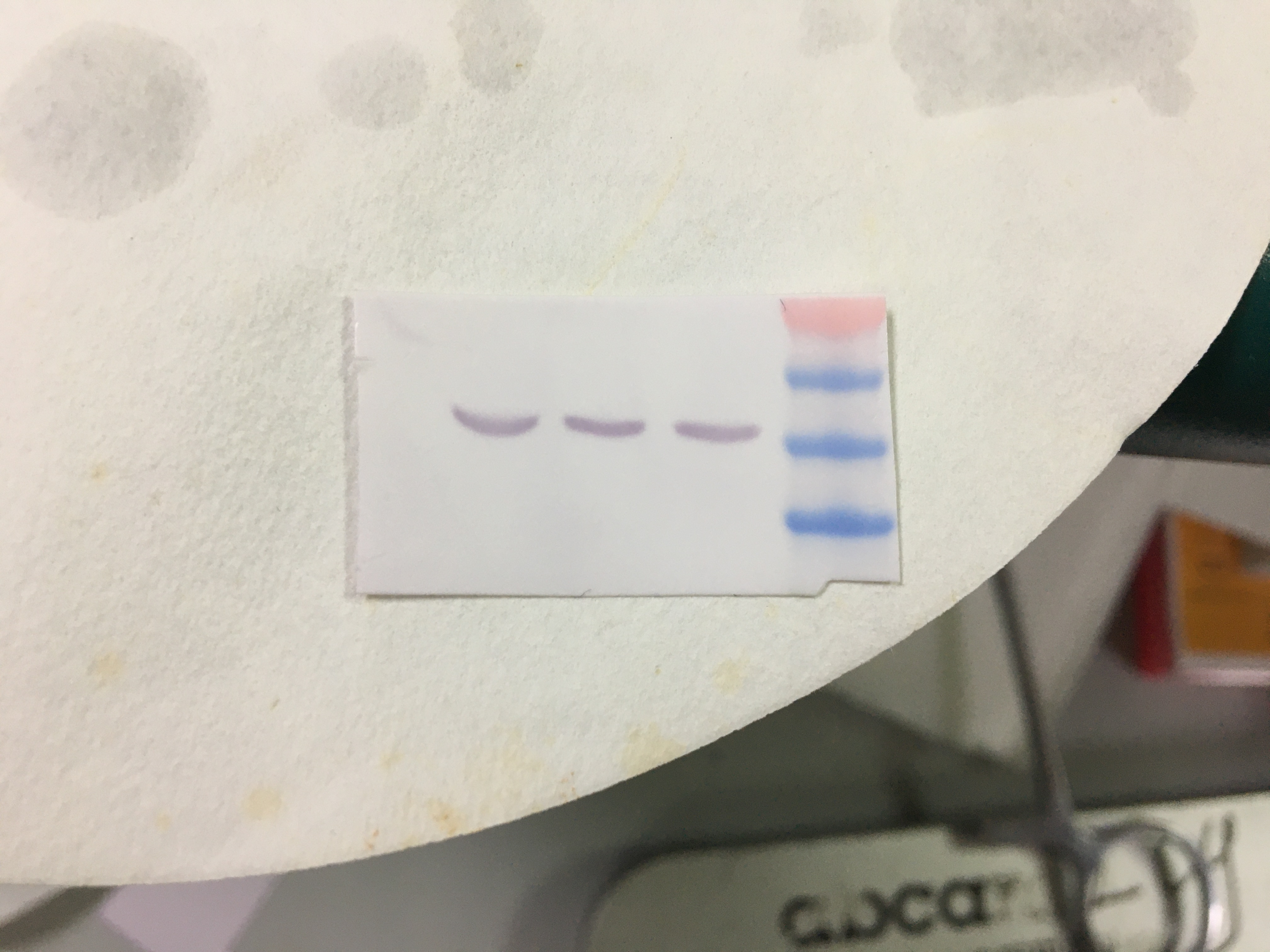

Supplement: Supplementary file 1 [file biology-15-00040-s001.zip › biology-4020702-supplementary/File S1/Figure5B-actin.jpeg]

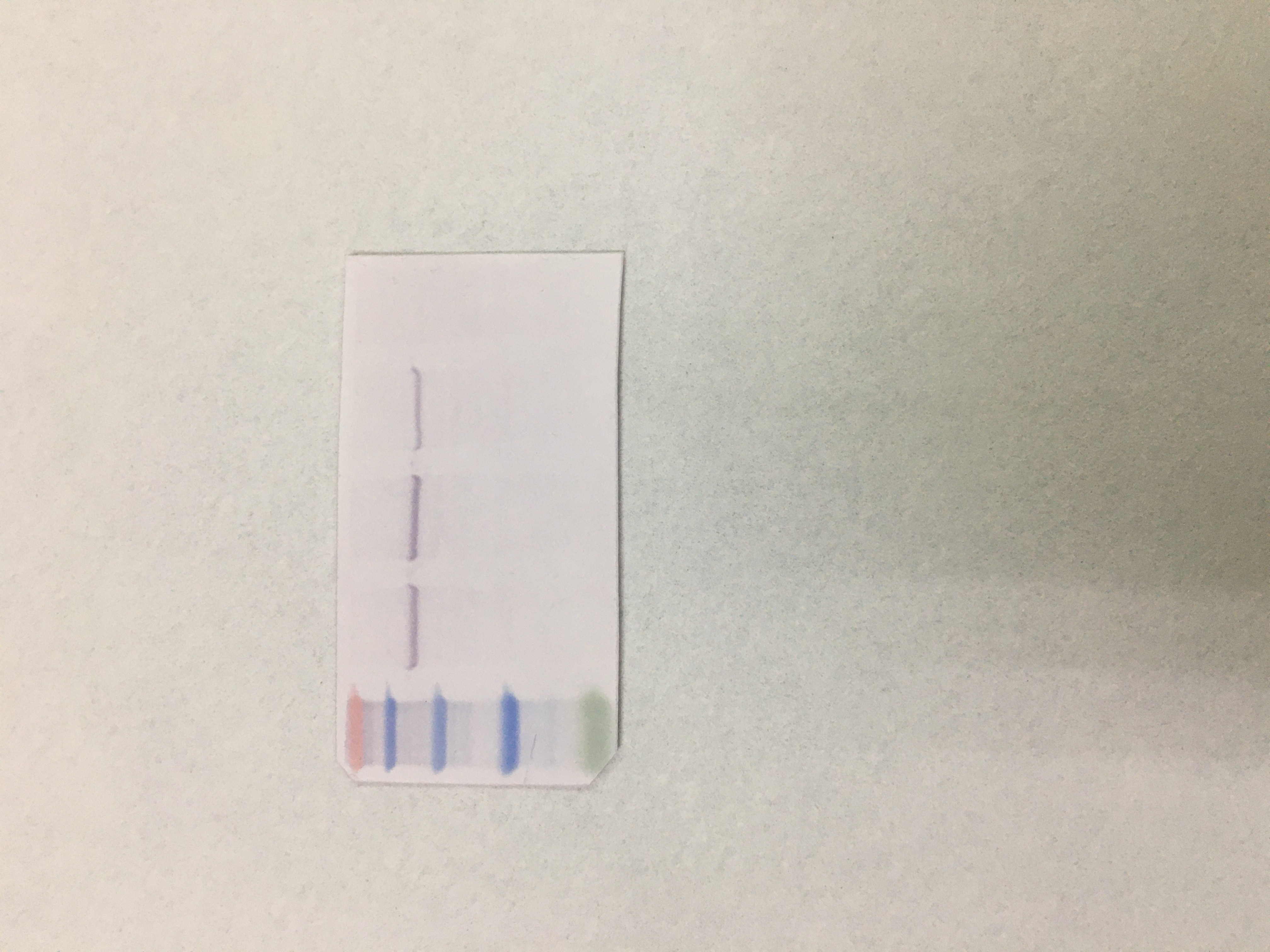

Supplement: Supplementary file 1 [file biology-15-00040-s001.zip › biology-4020702-supplementary/File S1/Figure5B-DHCR24.jpeg]

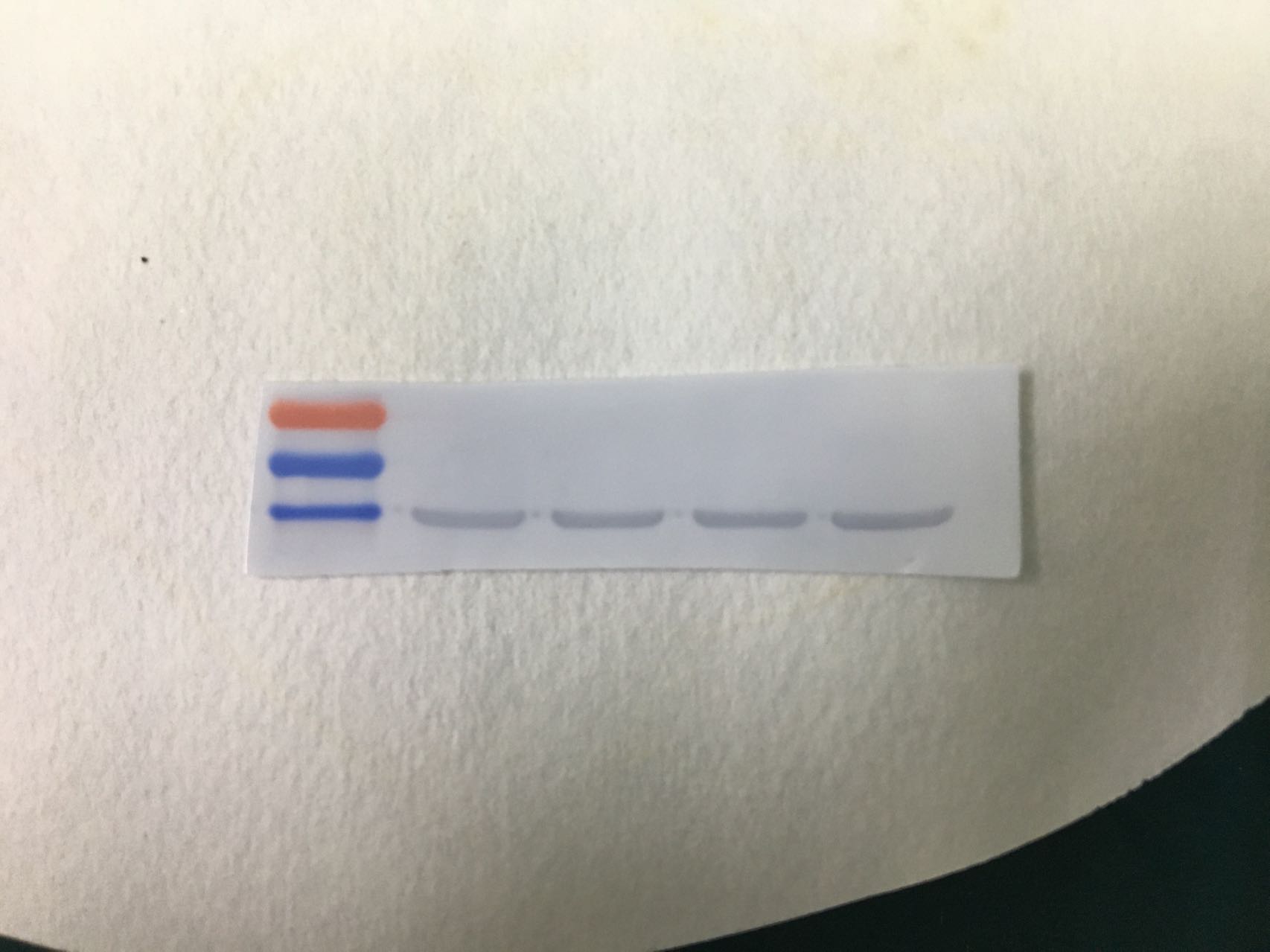

Supplement: Supplementary file 1 [file biology-15-00040-s001.zip › biology-4020702-supplementary/File S1/Figure7C-actin.JPG]

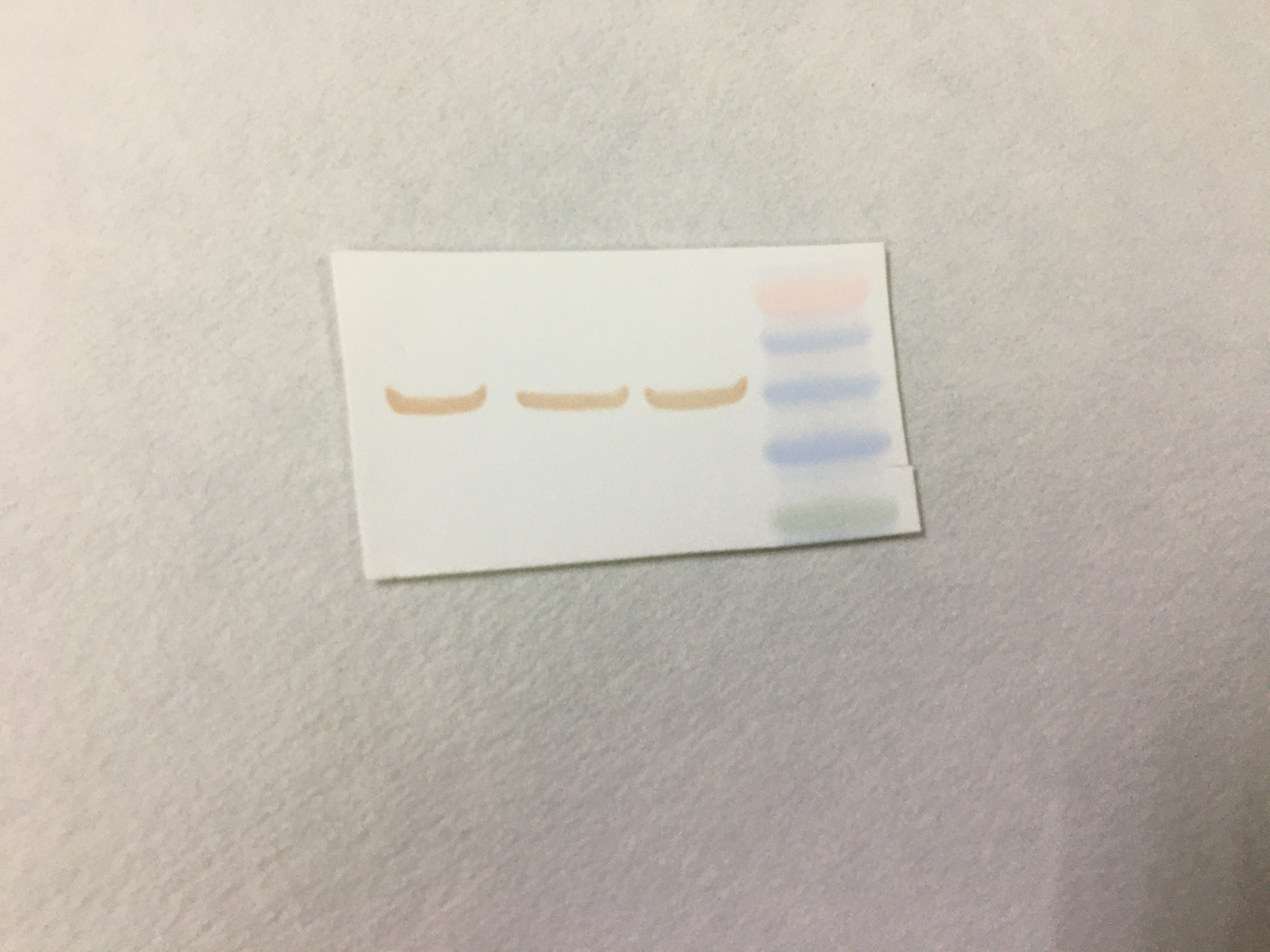

Supplement: Supplementary file 1 [file biology-15-00040-s001.zip › biology-4020702-supplementary/File S1/Figure7C-actin(NEW).jpeg]

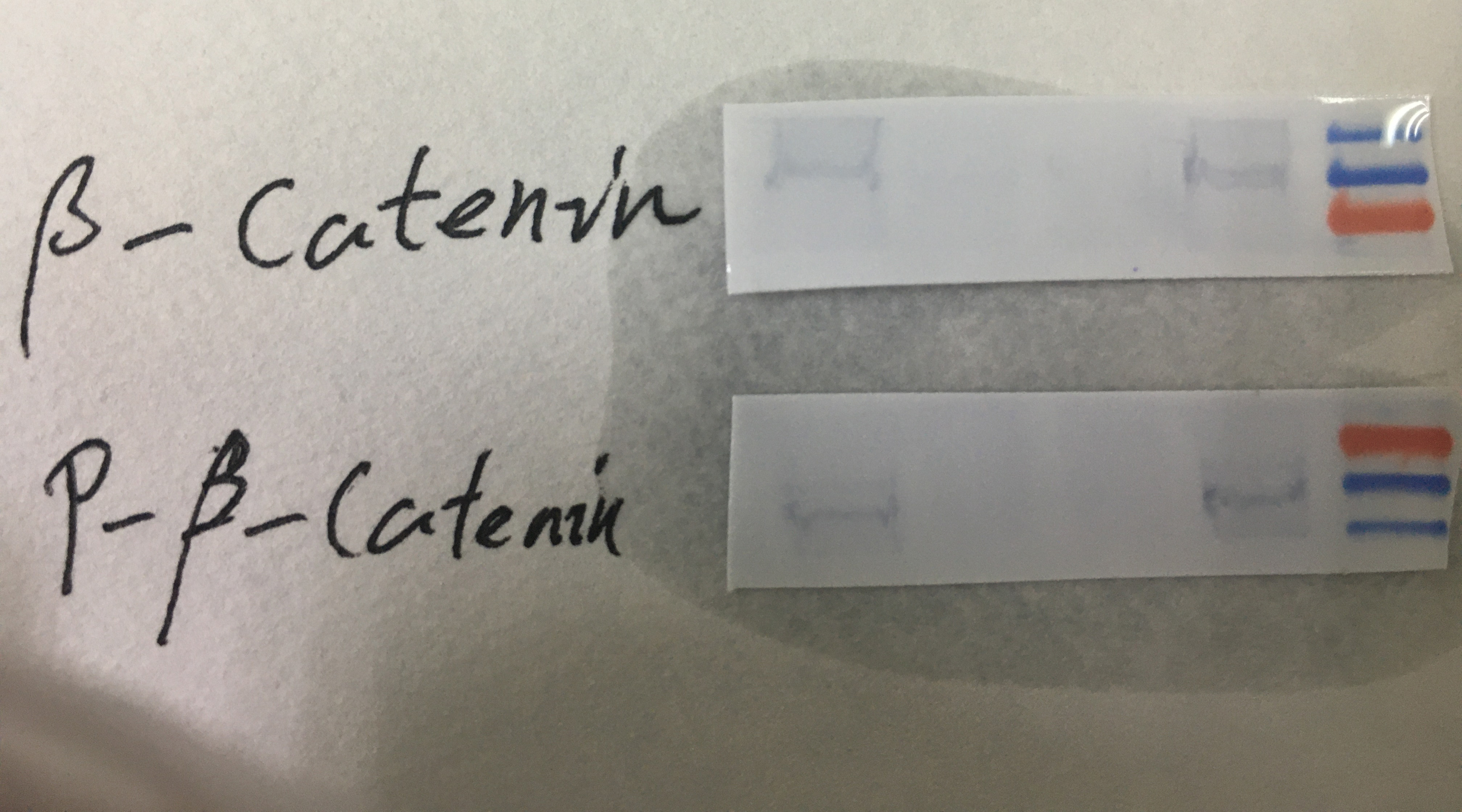

Supplement: Supplementary file 1 [file biology-15-00040-s001.zip › biology-4020702-supplementary/File S1/Figure7C-B-catenin and P-B-catenin.JPG]

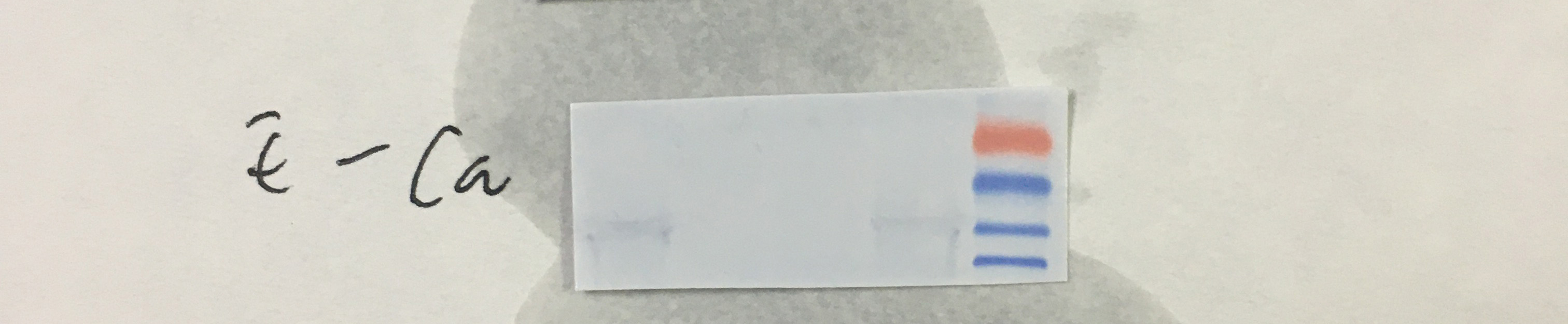

Supplement: Supplementary file 1 [file biology-15-00040-s001.zip › biology-4020702-supplementary/File S1/Figure7C-E-Ca.JPG]

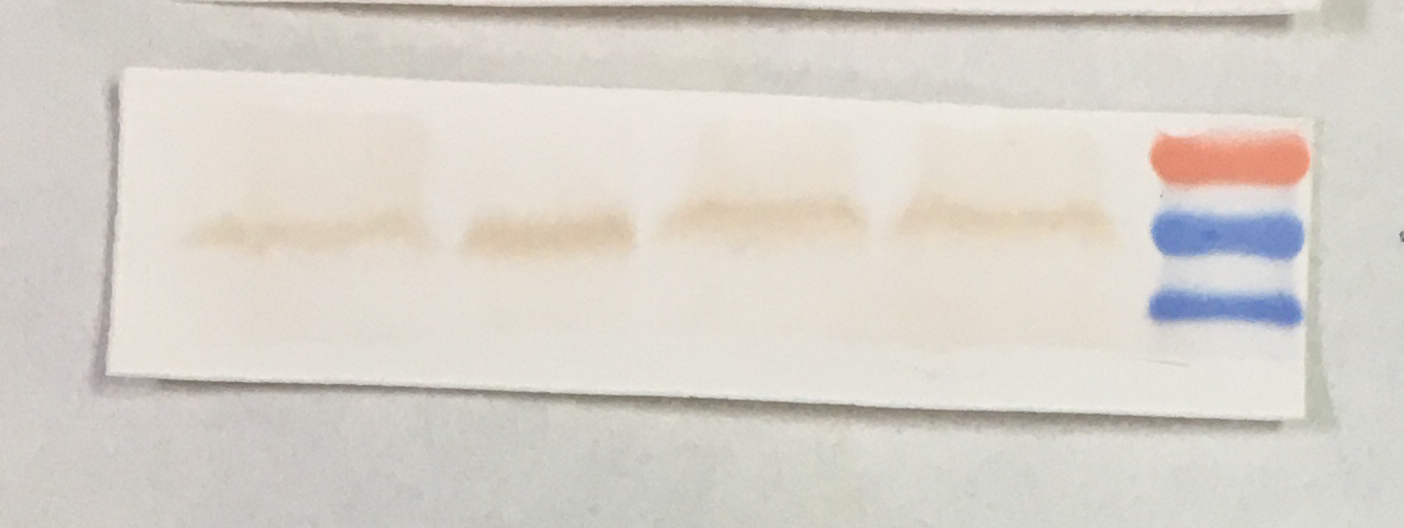

Supplement: Supplementary file 1 [file biology-15-00040-s001.zip › biology-4020702-supplementary/File S1/Fiugre7C-Vimintin(NEW).tif]
